# Transcriptome and long noncoding RNA sequencing of three extracellular vesicle subtypes released from the human colon cancer LIM1863 cell line

**DOI:** 10.1038/srep38397

**Published:** 2016-12-05

**Authors:** Maoshan Chen, Rong Xu, Hong Ji, David W. Greening, Alin Rai, Keiichi Izumikawa, Hideaki Ishikawa, Nobuhiro Takahashi, Richard J. Simpson

**Affiliations:** 1 Department of Biochemistry and Genetics, La Trobe Institute for Molecular Science (LIMS), La Trobe University, Melbourne, Victoria, Australia; 2 Department of Applied Biological Science, Graduate School of Agriculture, Tokyo University of Agriculture and Technology, Tokyo, Japan; 3 Global Innovation Research Organisation, Tokyo University of Agriculture and Technology, Tokyo, Japan

## Abstract

Previously we reported that LIM1863 colorectal cancer (CRC) cells secrete three distinct extracellular vesicle subtypes – two subpopulations of exosomes (apical EpCAM-Exos and basolateral A33-Exos) and shed microvesicles (sMVs) – with distinct protein and miRNA signatures. Here, we extend our *omics* approach to understand the fundamental role of LIM1863-derived EVs by performing a comprehensive analysis of their mRNAs and long non-coding RNAs (lncRNAs) using RNA-Seq. We show that 2,389 mRNAs, 317 pseudogene transcripts, 1,028 lncRNAs and 206 short non-coding RNAs selectively distributed to (i.e., are enriched in) LIM1863 EVs, relative to the parent cell. An Ensembl/UniProtKB analysis revealed 1,937 mRNAs encode canonical proteins, 348 isoforms (including splice-variant proteins), and 119 ‘missing proteins’ (i.e., annotated in Ensembl but not UniProtKB). Further dissection of our protein/RNA data revealed that 6/151 observed RNA binding proteins have the potential to interact with ~75% of EV-enriched RNAs. Intriguingly, the co-existence of U1 and U2 ribonucleoproteins and their cognate snRNAs in LIM1863 EVs suggests a possible association of CRC EVs with recipient cell splicing events. Our data reveal several potential lncRNA CRC biomarkers and novel splicing/fusion genes that, collectively, will advance our understanding of EV biology in CRC and accelerate the development of EV-based diagnostics and therapeutics.

Extracellular vesicles (EVs) are a heterogeneous population of endogenous nano- membranous vesicles that play a seminal role in intercellular communication by transferring biological information such as proteins, RNA species, DNA and lipids between cells^1^. EVs range in diameter from 50–1500 nm and can be classified into three broad classes based upon their protein/RNA profiles as well as biogenesis pathways: exosomes (50–120 nm), shed microvesicles (sMVs, 50–1500 nm, also referred to as microvesicles and microparticles), and apoptotic bodies. sMVs and exosomes arise from different biogenesis mechanisms, with sMVs originating by direct budding from plasma membranes, while exosomes have endocytic origins and are formed as intraluminal vesicles (ILVs) by inward budding of the limiting membrane of multivesicular bodies (MVBs); MVBs traffic to and subsequently fuse with the plasma membrane and release their sequested ILV contents into the extracellular environment as exosomes^1^. On the other hand, apoptotic bodies are released through outward budding and fragmentation of the plasma membrane of apoptotic cells. Other large vesicles such as oncosomes^2^,^3^ and migrasomes^4^ have been recently described, however their biogenesis is unclear.

In our ongoing studies aimed at understanding the physiopathological role of EVs in colorectal cancer (CRC) and their possible role as a source of blood-based diagnostic/prognostic markers for the disease we previously described robust procedures for isolating EVs from LIM1215^5^, SW480/SW620^6^, and LIM1863^1^,^7^,^8^ CRC cell lines. In the case of LIM1863 cells we showed that two distinct populations of exosomes as well as sMVs are released from these highly-polarised cells^8^. The sMVs were prepared from cell conditioned medium by differential centrifugation (10,000 *g*) and exosomes by sequential immunocapture using anti-A33 (A33-Exos) and anti-EpCAM (EpCAM-Exos) coupled magnetic beads. GeLC-MS/MS revealed that the protein profiles of the three EV subtypes were clearly distinguishable from each other^8^. This study showed that classical apical trafficking molecules such as CD63 (LAMP3), mucin 3, the apical intestinal enzyme sucrose isomaltase, dipeptidyl peptidase IV, and the apically-restricted pentaspan membrane glycoprotein prominin-1 (CD133) selectively distribute to EpCAM-Exos. In marked contrast A33-Exos are selectively enriched with classical basolateral trafficking molecules such as early endosome antigen 1 (EEA1), the Golgi membrane protein ADP-ribosylation factor and clathrin. While both exosome populations are CD81^+^/CD9^+^/CD44^+^, A33-Exos are CD63^-^. These findings are consistent with EpCAM- and A33-Exos being released from the apical and basolateral cell surfaces, respectively. Interestingly, the protein profile of LIM1863-derived sMVs bore little relation to that of the two exosome populations^8^ but, in stark contrast, are enriched in actin/microtubule network proteins, the centraspindlin motor complex proteins Kif23 and Racgap1^9^ and ESCRTIII subunits. The latter observations concur with sMVs isolated by sequential centrifugal ultrafiltration^10^.

To further define these LIM1863 EV subtypes, we investigated their microRNA (miRNA) expression profiles using small RNA-Seq (Illumina platform)^11^. This study revealed 254 cellular miRNAs of which 63 selectively distribute to the EVs, the most prominent being miR-19a/b-3p, miR-378a/c/d and miR-577 and members of the let-7 and miR-8 families. Let-7a-3p^∗^, let-7f-1-3p^∗^, miR-451-a, and miR-374-5p^∗^, mir-4454 and miR-7641 are common to all three EV subtypes. Six miRNAs (miR-320a/b/c/d, miR-3p, and miR-200c-3p) allow discrimination of LIM1863-derived exosomes from sMVs, while miR-98-5p was observed enriched in sMVs only. Of the EVs, A33-Exos contained the largest number of enriched miRNAs (32) of which 14 have not been previously reported in the context of CRC tissue/biofluid analyses^11^.

In this study, we extended our integrated *omics* analysis of LIM1863 CRC cell-released EVs and conducted a comprehensive analysis of mRNAs and lncRNAs in A33-/EpCAM-Exos and sMVs by using RNA-Seq. The goals of the study were to determine which coding transcripts (canonical mRNAs, isoform mRNAs, and pseudogene) and ncRNAs selectively distribute to the two LIM1863-derived exosome populations and to sMVs. We also examined so-called ‘missing’ protein transcripts – i.e., those annotated in Ensembl but not UniProtKB. We also correlated RNA binding proteins (RBPs) and ribonucleoproteins (RNPs) observed in these EV subtypes at the proteome level^8^ with possible cognate RNAs we identified at the RNA level. This integrated *omics* approach may provide a better understanding of the molecular and cellular events associated with EVs released from the human colorectal cancer cell line LIM1863 and possible role of EVs in splicing/ribosome biogenesis. Many of the lncRNAs observed in this study have not been reported in the context of CRC and warrant further investigation as possible diagnostic/prognostic biomarker candidates for the disease.

## Results and Discussion

### RNA sequencing and identification of LIM1863 mRNA and ncRNAs that differentially distribute to extracellular vesicles

**Figure 1:**
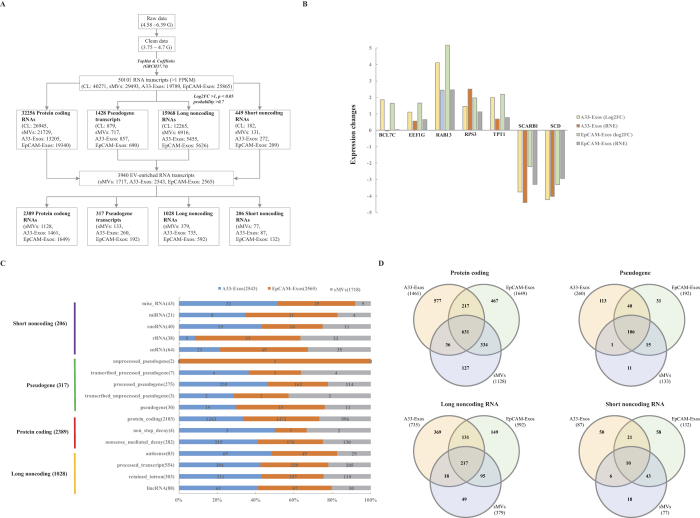
Gene expression profiling and number of RNA transcripts that preferentially distribute to extracellular vesicle subtypes released from colorectal cancer cell line LIM1863. (**A**) TopHat2 and Cufflinks2 identified a total of 50,101 RNA transcripts (>1 FPKM) in LIM1863 cells and released EVs. Using the statistical criteria Log_2_FC > 1, p-value < 0.05 and probability >0.7, a total of 3,940 RNA transcripts are enriched in EV subtypes (sMVs, A33-Exos, EpCAM-Exos) compared to CL. These transcripts are further annotated into four RNA classes: protein coding, pseudogene, short noncoding and long noncoding. (**B**) qRT-PCR validation of 7 RNA transcripts. (**C**) Number of short noncoding, long noncoding, pseudogene and protein coding RNA transcripts enriched in sMVs, A33-Exos and EpCAM-Exos. (**D**) Venn diagrams of protein coding, pseudogene, long noncoding and short noncoding RNA transcripts significantly enriched in sMVs, A33-Exos and EpCAM-Exos.

Extracellular vesicles comprise three main classes – exosomes, shed microvesicles (sMVs or microparticles) and apoptotic bodies^1^,^12^. Previously we reported that sMVs and two distinct populations of exosomes are released from the highly polarised LIM1863 colon carcinoma cell-derived organoids^8^; based on their protein profiles, the two exosome subtypes are consistent with one originating from the apical surface(EpCAM-Exos), the other (A33-Exos) from the basolateral surface^8^. Because the three EV types have distinct protein profiles, based on GeLC-MS/MS^8^, and miRNA profiles^11^, based on small RNA sequencing analysis, we surmised that cellular long RNA species (mRNA and lncRNA) might also be selectively enriched in these EVs. Exosomes and sMVs were purified using sequential immunocapture^8^ and consisted of vesicles ranging in size from 50–120 nm for exosomes and 50–1500 nm for sMVs^8^,^10^. The integrity of these EV preparations was further assessed by transmission electron microscopy and western blot analysis for the presence of exosomal (CD63, CD81, CD82, Alix, Tsg101) and sMV (Kif23) markers^10^,^11^. Next, we prepared cDNA libraries for large RNAs from parental LIM1863 cells (whole cell lysates, CL) and LIM1863 cell-derived sMVs and A33-/EpCAM-Exos^11^. Transcriptome data for these 4 samples (EV samples were pooled from over 400 individual culture media collections) yielded 4.58 to 6.39 G raw data (50.8 to 71 million reads) and 3.75 to 4.7 G clean data (41.7 to 52.2 million reads). Clean reads were aligned to the human genome sequence (version GRCh37.74) using TopHat2 and gene expression was profiled using Cufflinks2^13^ and matched human genome annotation provided by Ensembl (http://www.ensemble.org). Of the 50,101 mRNA/ncRNAs (>1 fragments per kilobase of transcript per million fragments mapped (FPKM) in at least one library) identified across all samples (40,271 for CL, 29,493 for sMVs, 19,789 for A33-Exos, and 25,865 for EpCAM-Exos), see Fig. 1A, 3,940 were significantly enriched in EVs relative to CL (1,717 sMVs, 2,543 A33-Exos, 2,565 EpCAM-Exos transcripts) using the following criteria: log_2_FC (fold change) >1, p-value < 0.05 and probability >0.7 (Supplementary Dataset). To validate the RNA-Seq results, 7 genes (BCL7C, EEF1G, RAB13, RSP3, TPT1, SCARB1, and SCD) were chosen for quantitative real-time PCR (qRT-PCR) (Fig. 1B). The selected mRNAs were significantly enriched, positively or negatively, in at least one comparison group. Three genes (CKS1B, GAPDH, and MFL2) present in CL and all EVs were used as internal controls for normalisation of qRT-PCR data. The results showed that expression patterns for these genes were in excellent agreement with the RNA-Seq findings.

### Annotation of EV-enriched protein coding RNAs

**Figure 2:**
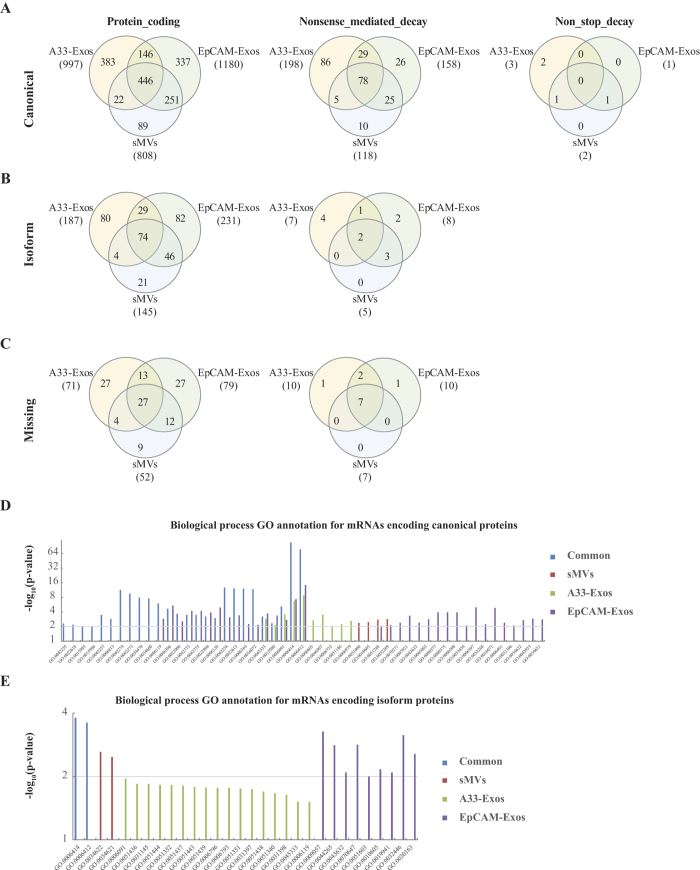
Annotation of EV-enriched protein coding RNA transcripts using UniProt and GO databases. (**A**) Canonical transcripts. (**B**) Isoform transcripts. (**C**) Missing protein transcripts (i.e., protein annotations seen in Ensembl but not UniProtKB). Ensembl/GENCODE analysis further categorized EV-enriched mRNAs (relative to CL) into protein coding (left), and those predicted to be targets of nonsense-mediated decay (middle) and non-stop decay (right). DAVID Bioinformatics Resource was used to annotate biological process GO for EV-enriched transcripts common to all EV subtypes and mRNAs encoding (**D**) canonical and (**E**) isoform proteins selectively distributed into EV subtypes.

The Ensembl Automatic Gene Annotation System^14^ (http://www.ensemble.org) and GENCODE^15^ were employed to annotate the 3,940 RNA transcripts enriched in LIM1863-derived EVs, relative to the parent cell. Of these, 2,389 are protein-coding mRNAs, 1,028 lncRNAs, 206 short noncoding RNAs (sncRNAs) and 317 are pseudogene-derived transcripts (Fig. 1C). Interestingly, Ensembl/GENCODE analysis revealed that the 2,389 protein-coding RNAs list contains 282 and 4 mRNAs predicted to be targets of nonsense-mediated decay (NMD)^16^ and non-stop decay^17^, another mRNA surveillance pathway, respectively. The distribution of the 2,389 protein-coding mRNAs in the EV subtypes revealed 631 to be common to all EVs while 577, 467, and 127 are significantly enriched, relative to CL, in A33-Exos, EpCAM-Exos and sMVs, respectively (Fig. 1D). In the next phase of our annotation strategy we focussed on those protein-coding mRNAs in the 2,389 dataset which were common to both Ensembl^18^,^19^ and UniProtKB/Swiss-Prot/TrEMBL^20^,^21^,^22^ databases. This annotation process identified 1,937 transcripts encoding canonical proteins, 348 transcripts encoding protein isoforms (including splice-variant proteins), and an additional 119 transcripts encoding proteins annotated in the Ensembl, but not the UniProtKB database – we refer to the latter as ‘missing’ proteins (i.e., gene-encoded proteins where there is no confirmatory protein/peptide information). Further analysis of the 1,937 canonical protein dataset revealed 1,674 protein-coding transcripts, 259 NMD transcripts and 4 predicted to be the target of non-stop-decay (Fig. 2).

The distribution pattern of gene-encoded canonical proteins in the EV subtypes showed 446 encoded proteins common to the three EV subtypes, 383 selectively enriched in A33-Exos relative to CL, 337 in EpCAM-Exos and 89 selectively enriched in sMVs (Fig. 2A). To gain insight into the function of the 446 cellular canonical mRNAs that are selectively enriched in three EV subtypes we performed a Gene Ontology (GO) analysis. Interestingly, GO terms were related to ‘translation’ (GO:0006412), ‘ribosome biogenesis’ (GO:0042254), and ‘rRNA processing’ (GO:0006364) in the biological process category (Fig. 2D). These significantly enriched GO terms (*p* < 0.01) common to all LIM1863 EVs indicate the possibility of a hitherto, unrecognized role of EVs in cell-cell communication, especially in protein translation-related processes such as ‘ribosome biogenesis for energy metabolism’ and ‘cellular growth’ – processes which are of central importance and considered as hallmarks of cancer^23^,^24^. These GO terms were also prominent in selectively-enriched canonical mRNAs in A33-Exos and EpCAM-Exos (Fig. 2D); in contrast, for sMVs the GO terms ‘negative regulation of cellular protein metabolic process’ (GO:0032269) and ‘negative regulation of protein modification process’ (GO:0031400) are exclusively represented in the biological processing category (Fig. 2D). The most significant KEGG pathways for commonly enriched mRNAs and selectively enriched mRNAs in A33-Exos and EpCAM-Exos were ‘ribosome’ (hsa03010) and ‘ribosome’ (hsa03010)/’spliceosome’ (hsa03040), respectively (Supplementary Table S1). These GO/KEGG pathway analysis findings imply that the sMVs, A33-Exos and EpCAM-Exos may have different functional roles in recipient cells.

An inspection of the GO terms showed that the most prominent transcripts enriched in all EV subtypes, relative to parental LIM1863 cell lysates, were ribosomal protein mRNAs encoding 37 and 52 proteins in the small (40 S) and large (60 S) ribosome subunits (Supplementary Dataset). Other significantly enriched transcripts common to all EV subtypes were tumour protein, translationally-controlled 1 (TPT1), the EEFs (eukaryotic translational elongation factors EEF1G, EEF1B2, EEF1D and EEF2), FTL (ferritin light polypeptide), RAB13, the transcriptional elongation factors TCEB1/TCEB2, and 13 transcripts encoding cellular proteins associated with the mitochondrial complex.

We next looked at prominent canonical transcripts differentially distributed in the individual EV subtypes compared to CL. There are three protein classes potentially transcribed by these mRNAs that stand out – eukaryotic translation initiation factors (EIFs), the heterogeneous nuclear riboproteins (HNRNPs), and mitochondrial ribosomal proteins (MTRPs). For example, A33-Exos contain 6 EIFs (EIF-3E, -3L, -4A1, -2AK1, -4E, and EIF5E), one HNRNP (HNRPM), and 16 MTRPs (MRPL-21, -55, -42, -30, -49, -48, -36, -13, -48, -37, -53, -1, MRPS-28, -14, -17, -35, -18C, and -18C) not seen in sMVs and EpCAM-Exos. In contrast, EpCAM-Exos there are 6 EIFs (EIF-3F, -3E, -3K, -3M, 4EBP1, and -1), 6 HNRNPs (HNRNP-A1, -A3, -C, -PH1, and -PL), and 8 MTRPs (MRPL-33, -22, -13, -51, -18, 53, -48, and MRPS36) exclusive to this EV. In the case of sMVs enrichment of 3 EIFs (EIF-3K, -3M and -2B4), two HNRNPs (HNRNPC and HNRNPH1) and three MTRPs (MRPL-52, -21 and -12) are selectively enriched in this EV subtype. For a summary of these data, see Supplementary Dataset.

We next wanted to look at potential protein isoform sequences – i.e., protein products generated by alternative splicing, alternative promoter usage and alternative translation initiation^22^. A total of 336 mRNAs encoding isoform proteins were observed to be enriched (when compared to CL) in EVs released from LIM1863 cells – of which 74 are common to all EV subtypes and 21, 80, and 82 differentially distribute in sMVs, A33-Exos and EpCAM-Exos, respectively (Fig. 2B, Supplementary Dataset). TPT1 and a large group of ribosomal protein transcripts (RPL12, RPL13, RPL17, RPL18, RPL28, RPL31, RPLP0, RPLP1, RPS24 and RPS29) are common to all EV subtypes, while two IL32 isoform transcripts (ENST00000440815 and ENST00000530890) are specifically enriched in sMVs, three mRNAs encoding isoforms of small EDRK-rich factors (SERF1A, SERF1B and SERF2), and 4 splice variant transcripts for transmembrane proteins (TMEM126B, TMEM134, TMEM14B and TMEM54) preferentially distribute to EpCAM-Exos. Using the DAVID Bioinformatics Resource^25^,^26^ 51 (common), 17 (sMVs), 56 (A33-Exos), and 59 (EpCAM-Exos) of these enriched splice variant transcripts were found to be recognized in the GO biological process category (Fig. 2E, Supplementary Table S2). Those splice variant transcripts common to all EV subtypes are mainly involved in the biological processes of ‘translational elongation’ (GO:0006414) and ‘translation’ (GO:0006412). Interestingly, splice variant mRNAs that selectively distribute to sMVs, A33-Exos and EpCAM-Exos are found in different GO terms – for example, sMVs (‘cellular macromolecular complex assembly’ (GO:0034622) and ‘subunit organization’ (GO:0034621)), EpCAM-Exos (‘macromolecule catabolic process’ (GO:0009057) and ‘protein modification by small protein conjugation’ (GO:0032446)); no significant biological process GO terms (p-value < 0.01) were observed for isoform mRNAs enriched in A33-Exos. These observations point to different functional roles of LIM1863 EV subtypes in recipient cells.

Interestingly, of the 119 EV-enriched mRNAs observed in Ensembl but ‘missed’ in UniProtKB 27 are common to all the EV subtypes 9, 27 and 27 selectively distribute to sMVs, A33-Exos and EpCAM-Exos, respectively (Fig. 2C, Supplementary Dataset). Significant GO Biological Process annotations were found for transcripts common to all EV subtypes and those selectively enriched in EpCAM-Exos. For example, two transcripts (ENST00000439403 and ENST00000372099, GTF3A and GTF3C5) probably function in “rRNA transcription” (GO:0009303) and “transcription from RNA polymerase III promoter” (GO:0006383), while ENST00000444743 and ENST00000433710 (TIMM23 and MIPEP) are involved in ‘protein transport and targeting in mitochondria’. Next we asked how many of these 119 missed transcripts might originate from the same genes encoding canonical/isoform proteins. This analysis revealed **63/119** transcripts not originating from these genes (Supplementary Table S3) – of these the most prominent transcript (ENST00000446260) encodes an uncharacterised protein from the chromosome1 open reading frame 122 (C1orf122 gene). Needless to say, further dissection of these 63 missing transcripts not seen in UniProtKB may accelerate the completion of the Human Proteome Project^27^,^28^,^29^.

### Novel alternative splicing events and fusion genes

Increasing evidence indicates that missense mutations and unique cancer-derived fusion transcripts are potential sources of neoantigens, which could provide a valuable source of disease biomarker and novel therapeutic approaches^30^,^31^. Using TopHat2 we identified 268 novel alternative splicing events (Supplementary Table S4) and 33 fusion genes were identified using Software ChimeraScan version 0.4.5 (Supplementary Table S5). To increase the reliability of these searches we set stringent criteria – >100 fragments in the case of alternative splicing sites and >10 fragments for fusion gene connection coverage. Although none of the identified fusion genes have been reported in CRC, three have been reported in other cancers - SH3D19/LRBA in primary myelofibrosis^32^; RIPK2/OSGIN2 in primary urethral clear-cell adenocarcinoma^33^; and GOLT1A/KISS1 in bladder cancer^34^. Interestingly, most of the alternative spicing events occurred in ribosomal protein and TPT1 (TCTP) genes, whose normal transcripts are also highly expressed and enriched in the EVs (see above). While we do not see any splice variant forms of ribosomal or TCTP proteins in our datasets it is interesting to speculate that the corresponding transcripts we observe in EVs may be translated upon EV uptake in recipient cells; this, in turn, may contribute to onset of apoptosis and cancer, as described elsewhere^35^,^36^,^37^.

### Annotation of transcripts derived from pseudogenes

**Figure 3:**
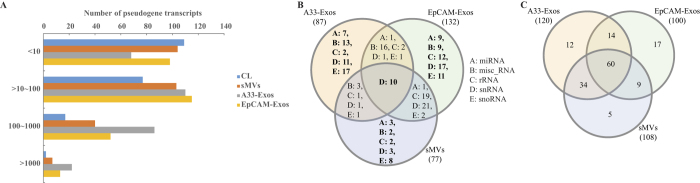
Noncoding RNA transcripts and RNA binding proteins enriched in LIM1863 cell-derived EVs. (**A**) Distribution of the expression levels of EV-enriched pseudogene transcripts. (**B**) Venn diagram of EV-enriched short noncoding RNAs. (**C**) Venn diagram of RNA binding proteins identified in LIM1863 cell-derived EVs.

Because the role of pseudogenes in physiology and disease is gaining importance^38^,^39^ we next asked whether there are any pseudogene transcripts in LIM1863-derived EVs. Using the same criteria for protein-coding mRNAs, a total of 317 pseudogene transcripts were found to be enriched (>100 FPKM) in EVs released by LIM1863 cells, especially in A33-Exos (Fig. 3A, Supplementary Dataset). Common EV-enriched pseudogene transcripts include 105 processed and one unprocessed pseudogene transcripts. Of these, the 10 most highly-expressed pseudogene transcripts are RPL41P1, RPL39P3, EEF1A1P5, CTD-2031P19.4, CTB-63M22.1, RP11-742N3.1, RP11-122C9.1, RPL9P8, RPL9P9 and RP11-466H18.1. Eleven processed pseudogene transcripts preferentially distribute to sMVs (MRPS10P1, TECRP1, ATP5EP1, RP11-158M2.6, POLR2KP1, CTD-2218G20.1, CTD-3141N22.1, RP11-486A14.1, AL049542.1, PSMC1P5 and AC012615.1), 113 in A33-Exos and 31 in EpCAM-Exos. Of note, 67 of the 113 pseudogene transcripts specifically enriched in A33-Exos were ribosomal protein pseudogenes, 4 derived from eukaryotic translation elongation factor 1 alpha 1 gene, and 3 were from ferritin, heavy polypeptide 1 gene.

### Annotation of ncRNA data

It is now apparent that the vast majority of the genome is transcribed as non-protein-coding RNA (ncRNA)^40^ and that many of these ncRNAs are of crucial importance for normal development and physiology and for disease^41^,^42^. In this study, we identified 206 sncRNAs and 1,028 lncRNAs preferentially distributed to LIM1863 cell-derived EVs (Fig. 1C). In the case of sncRNAs we see 10 cellular sncRNAs enriched in all three EV subtypes and 18, 50 and 58 that selectively distribute to sMVs, A33-Exos and EpCAM-Exos, respectively. Detailed category information (miRNA, misc_RNA, rRNA, snRNA and snoRNA) for the EV-enriched sncRNAs is given in Fig. 3B and Supplementary Dataset.

#### MiRNAs

The miRNAs seen in this study were either primary miRNA transcripts (pri-miRNAs) or miRNA precursors (pre-miRNAs). Four EV-enriched pre-miRNAs (MIR3661, MIR941-1, MIR1282 and a novel miRNA AC009065) identified in sMVs were highly expressed (>100 FPKM), of them MIR3661 (ENST00000577394) was shared with EpCAM-Exos. In A33-Exos, we found two known pre-miRNAs (H19 and MIR1182) and five novel pre-miRNAs. Interestingly, H19 encodes miR-675 which regulates tumour suppressor RB in human colorectal cancer^43^. EpCAM-Exos was identified to contain MIR671 and another 8 novel miRNAs (for a detailed list of identified pri-miRNAs, see Supplementary Dataset).

#### Misc_RNAs

Several miscellaneous snRNAs (misc_RNA) were enriched (>100 FPKM) in LIM1863-derived exosomes (Supplementary Dataset), especially 7SL and Y_RNA transcripts. Interestingly, 7SL is an RNA component of the SRP (signal recognition particle), which associates with the ribosome and targets nascent proteins to the endoplasmic reticulum for secretion or membrane insertion^44^,^45^. Y_RNAs are small noncoding RNAs, which are components of Ro60 ribonucleoprotein particle, a target of autoimmune antibodies in patients with systemic lupus erythematosus^46^. They are also necessary for DNA replication through interactions with chromatin and initiation proteins^47^,^48^. In our study while we do not see canonical Y_RNA products (e.g., Y-RNAs 1,3,4, and 5) we do observe homologs of Y-RNA that bare RNA sequence similarities^49^; the function of this category of misc_RNAs it yet to be elucidated.

#### rRNAs

Ribosomal ribonucleic acid (rRNA), the RNA components of the ribosome is essential for protein synthesis in all living organisms. It constitutes the predominant material within the ribosome, which is approximately 60% rRNA and 40% protein by weight. In this study we identified 5 and 33 rRNA transcripts enriched specifically in A33-Exos and EpCAM-Exos, respectively. The number of rRNA transcripts specifically enriched in EpCAM-Exos (12) is 6 fold greater than that in A33-Exos (2) (Fig. 3C). It is interesting that rRNAs enriched in the LIM1863-derived exosomes and sMVs are 5 S rRNAs.

#### snRNAs

The snRNA (small nuclear ribonucleic acid) class of small RNA molecules found within the splicing speckles and Cajal bodies of the cell nucleus in eukaryotic cells^50^. Also referred to as spliceosome RNAs, the snRNAs are integral components of the spliceosome, a large ribonucleoprotein (RNP) made up of over 200 different proteins and five snRNAs - U1, U2, U4, U5 and U6^50^. snRNAs, the largest group of sncRNAs identified in this study are significantly enriched in LIM1863 released EVs; 35, 23 and 49 snRNA transcripts are enriched in sMVs, A33-Exos and EpCAM-Exos, respectively (Table 1).

#### snoRNAs

Small nucleolar RNAs (snoRNAs) are a class of snRNAs responsible for guiding a series of site-specific post-translational modifications of rRNAs, tRNAs and snRNAs^51^,^52^. There are two main types of snoRNAs – H/ACA box snoRNAs (direct modification of nucleoside uridine to pseudouridine) and the C/D box snoRNAs which directs methylation of nucleosides^51^. In total we identified 40 EV-enriched snoRNAs, in which 8, 17 and 11 selectively distribute to sMVs, A33-Exos and EpCAM-Exos, respectively (Table 2).

#### lncRNAs

In addition to the above, there is a further class of transcripts referred to as lncRNAs which are operationally defined as transcripts that are >200 nt in length and lack protein coding capability^53^. lncRNAs can be roughly classified as intergenic, intragenic/intronic, and antisense-based on their position relative to protein-coding genes^41^,^53^. In this study we identified 1,028 lncRNAs (Fig. 1C) comprising the following categories – 83 antisense lncRNAs, 554 processed transcripts, 303 retained-intron transcripts and 88 lincRNAs (long intergenic non-coding RNAs). Of these, 217 cellular lncRNAs were enriched in all three EV subtypes while 131 are enriched in both exosome types and 49, 369, and 149 preferentially distribute to sMVs, A33-Exos and EpCAM-Exos, respectively (Fig. 1D). Abundant antisense lncRNAs selectively enriched in both exosome subtypes include RP5-940J5.9, RP11-290D2.6, as well as 7 ZFAS1 (ZNFX1 antisense RNA 1) and 5 C17orf76-AS1 (FAM211A antisense RNA 1) isoforms. Interestingly, C17orf76-AS1 (LRRC75 antisense RNA1) is up-regulated in 5-fluorocil resistant CRC cell lines and is reported to regulate apoptosis^54^. We found several highly-enriched antisense lncRNAs (Log2FC > 2) common to all three EV subtypes (e.g., ZFAS1). Antisense lncRNA AC007193.8 uniquely distributed to sMVs, RUSC1-AS1, TM4SF1-AS1, DLGAP1-AS1 and DLGAP1-AS1to A33-Exos, while SETD5-AS1, DNAJC27-AS1 and TTC28-AS1 selectively distributed to EpCAM-Exos. Of these antisense lncRNA ZFAS1, which has been reported in breast cancer tissue^55^, liver cancer^56^ and CRC^57^, is thought to function as an oncogene by destabilisation of p53 and interaction through the CDK1/cyclin B1 complex leading to cell cycle progression and inhibition of apoptosis^57^.

In the case of lincRNAs, several EV-enriched lincRNAs attracted our attention because of their significance in cancer progression and diagnosis, such as small nucleolar RNA host genes (SNHG5, SNGH6 and SNHG8), growth arrest specific transcript 5 (GAS5), LINC00493, TP53 target 1 (TP53TG1), MIR4435-1 host gene (MIR4435-1HG) and H19. In the case of GAS5, its down-regulation is reported to be a poor prognosticator of cancers such as breast^58^, prostate^59^, gastric^60^, lung^61^, bladder^62^, colorectal^62^ and cervical^62^. GAS5 is reported to act by enhancing G1 cell cycle by regulating cyclin-dependent kinase 6 (CDK6)^63^. For a list of enriched antisense lncRNAs and lincRNAs in LIM1863-derived EVs, see Supplementary Dataset.

### Ribonucleoprotein (RNP) complexes associated with LIM1863-derived EVs

Because ncRNAs can function as molecular scaffolds to specify higher-order organisation in ribonucleoprotein (RNP) complexes and in chromatin states^64^,^65^ we next asked whether EV cargo includes RBPs and if any of our identified RNA transcripts could potentially bind to these RBPs (i.e., cognate binding partners).

**Figure 4:**
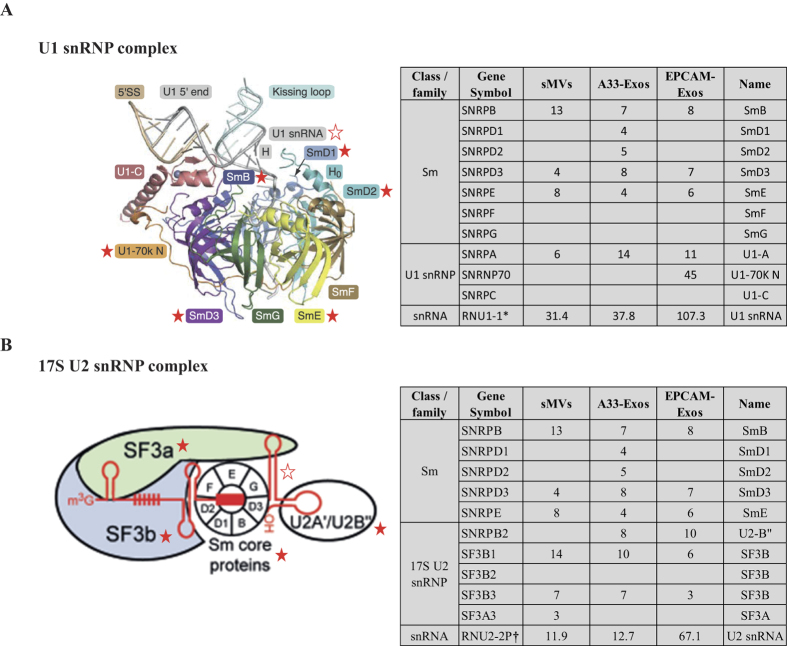
U1 and U2 complex related proteins and snRNAs identified in this study. (**A**) X-ray structure^86^ (left) (reproduced under a CC-BY license agreement (https://creativecommons.org/licences/by/4.0/)) of U1 snRNP complex revealed Sm proteins (marked with red solid five-pointed stars), U1 snRNPs (marked with red solid five-pointed stars) from MS/MS data and U1 snRNAs (marked with hollow five-pointed star) from this study may form pre-complexes in EVs. Significant peptide spectra mapping to these proteins and normalized gene expression for U1 snRNA (RNU1-1) the table (right panel). (**B**) In U2 snRNP complex^87^, and peptide spectra for proteins such as Sm, splicing factors and U2 snRNA (shown in the table in the right panel). *Major U1 snRNA transcript. †Major U2 snRNA transcript, also referred to RNU2 snRNA.

Highly-purified LIM1863-derived EVs were prepared using a combination of differential centrifugation (for sMVs) and sequential immunoaffinity capture (for A33-Exos and EpCAM-Exos), as described by Tauro *et al*.^8^,^66^. GeLC-MS/MS revealed a total of 151 RBPs and 71 ribosomal proteins (Supplementary Table S6), as defined^65^,^67^. For subsequent analysis the ribosomal proteins were not taken into consideration. It can be seen in the Venn diagram (Fig. 3C) that 12, 17, and 5 cellular RBPs selectively distribute to A33-Exos, EpCAM-Exos, and sMVs, respectively. Interestingly, of these 6 members of the heterogeneous nuclear RNA-binding proteins (hnRBPs)^68^, 16 eukaryotic initiation factor (eIF) family proteins^23^, 3 RNA helicases of DEAD box family (DDX)^69^ and 8 splicing factors, some of which (e.g., hnRNPA2B1) have been implicated in the sorting of RNAs into EVs^70^. Next we used StarBase (v2.0)^71^, which comprises >6000 entries and 111 CLIP-Seq experiments, to identify those RNAs and RBPs from our LIM1863 EV datasets that could potentially interact. Table 3 reveals that 7 of the 151 RBPs seen in our MS-based studies could bind to many of the RNAs we obtained by RNA-Seq. We also interrogated our data for evidence of RNP complexes of snRNAs and RBPs associated with spliceosome subunits. Our findings (Fig. 4) reveal evidence for the presence of two spliceosome complexes – the U1 and U2 subunits – that are critical for pre-mRNA processing^50^. To our knowledge this is the first report of EV-associated ribonucleoprotein particles.

### LIM1863 cancer-associated mRNAs and lncRNAs selectively distribute to EVs

Finally, we asked whether any of the RNA species identified in LIM1863-derived EVs might be implicated in CRC or other cancer types. As shown in Table 4 we list several EV-enriched mRNAs seen in our study (e.g., TPT1, several ribosomal protein (RP) genes, EEF1A1, EEF1B2 and FTL, and lncRNAs, such as SNHG5, SNHG6, SNHG7, SNHG8, ZFAS1, H19 and LINC00116) have also been reported to be up-regulated in tumour tissues. It is interesting to note that GAS5, which has been reported to be down-regulated in CRC tumour tissue^72^ as well as HCC^73^ and pancreatic cancer^74^, is highly expressed and significantly enriched in all LIM1863-derived EVs. Since GAS5 overexpression has been implicated in cell growth arrest and apoptotic induction^58^, it is speculated that its release from the cells via EVs might provide a mechanism for lowering its cellular concentration, in a manner similar to PTEN in glioblastomas^75^.

We used two CRC gene expression studies (SRP022054^76^ and SRP029880^77^) from the SRA database (https://www.ncbi.nlm.nih.gov/Traces/sra/sra.cg) - 4 and 18 pairs of tumour/normal tissues from CRC patients, respectively - to validate the expression levels of candidate mRNAs and lncRNAs found in our study. Heat maps for these two studies (Supplementary Fig. S1) revealed several RNAs species from our LIM1863 studies that are up-regulated in tumour biopsies that warrant further investigation as potential blood-based CRC biomarkers.

## Conclusion and Perspectives

Secretion and reciprocal exchange of EVs between cells is emerging as a central paradigm in cancer biology, especially in the tumor microenvironment. EVs contain protein/RNA/DNA/lipid-laden cargoes which upon uptake by recipient cells play a critical physiological role in healthy and pathological conditions. Previously, we reported the isolation of three EV subtypes from the human CRC cell line LIM1863 –sMVs and two exosome populations (basolateral A33-Exos and apical EpCAM-Exos). Although profiling their protein^8^,^10^ and miRNA^11^ cargoes has shed much light on the nature of these vesicles, identifying their RNA cargo will further assist our understanding of how they modulate recipient cell behavior. In this study we identified a total of 2,389 cellular protein coding RNAs, 317 pseudogene transcripts, 1,028 long noncoding RNAs and 206 short noncoding RNAs that preferentially distributed to LIM1863 EVs. For each RNA category we identified a number of RNA transcripts commonly enriched in all EV subtypes, relative to CL, as well those specifically enriched in each EV subtype. TPT1 transcripts, mRNAs encoding ribosomal proteins, FTL, and lncRNAs ZFAS1 and SNHGs, which are commonly enriched in the three EV subtypes, are up-regulated in previously published CRC tumor tissues^76^,^77^ when compared to matched normal colon tissue. Additionally, we observe several novel RNAs that warrant further analysis as potential CRC prognostic biomarker candidates and targets for clinical management. Among the protein coding RNAs, we found 446 mRNAs encoding canonical proteins including TPT1, ribosomal proteins, FTL and EEFs, 74 mRNAs encoding isoform proteins and 27 mRNAs without protein evidence in UniProtKB (i.e., ‘missing’ proteins). Novel alternative splicing and gene fusions we identified in LIM1863 cells and derived EVs warrant further study as possible neoantigen sources. Finally, we observed 151 RNA-binding proteins (RBPs) in LIM1863 EVs - 7 of which are reported to bind to RNA transcripts identified in our study by RNA-Seq. Remarkably, interrogation of the Spliceosome Database (http://spliceosomedb.ucsc.edu/) identified the RBPs and cognate snRNAs for the ribonucleoprotein complexes U1 and U2. To our knowledge this is the first report of RNPs in EVs and raises the possibility that EVs may play a key role modulating mRNA splicing upon uptake in recipient cells.

## Materials and Methods

All methods were carried out in accordance with the approved guidelines of La Trobe Institute for Molecular Science.

### Cell culture

LIM1863 cells^78^ were initially cultured in 175 cm^2^ flasks (Invitrogen, Carlsbad, CA) with RPMI-1640 supplemented by 5% foetal calf serum (FCS), 0.1% insulin-transferrin-selenium (ITS, Invitrogen), 100 U/ml penicillin and 100 μg/ml streptomycin at 37 °C and 5% CO_2_. Cells (~3 × 10^7^) were transferred into the Cultivation chamber of a CELLine CL-1000 Bioreactor classic flask (Integra Biosciences) and cultured at 37 °C and 5% CO_2_ atmosphere as previously described^11^. Culture medium was replaced twice a week and the cell suspension from the Cultivation chamber was harvested every 48 hr.

### EV purification

LIM1863 cells were cultured in serum-free medium supplemented with insulin-transferrin-selenium for 24 h, according to Ji *et al*.^11^. The culture medium (CM) was collected and subjected to differential centrifugation at 4 °C, first at 480 *g* for 5 min followed by 2,000 *g* for 10 min to remove intact cells and cell debris and then a final centrifugation step (10,000 *g*, 30 min) to isolate shed microvesicles (sMVs)^7^,^79^. The resulting supernatant was centrifuged at 100,000 *g* to harvest crude exosomes which were then fractionated into two distinct exosome subpopulations (A33-Exos and EpCAM-Exos) by sequential immunocapture using Dynabeads™ (Invitrogen) loaded with anti-A33 monoclonal antibodies^5^ in tandem with anti-EpCAM (CD326)-monoclonal antibody bound magnetic microbeads (Miltenyi Biotec), as described^8^.

### Protein Quantification

Protein quantification based on protein staining densitometry was performed by 1D-SDS-PAGE/SYPRO^®^ Ruby, as previously described^79^,^80^.

### Transmission electron microscopy (TEM)

A33-Exos and EpCAM-Exos subpopulations (1 μg/10 ml PBS) were applied for 2 min to 400 mesh copper grids coated with a thin layer of carbon. Imaging was performed using a JEOL JEM-2010 transmission electron microscope operated at 80 kV, as described^8^,^11^.

### Western blot analysis

sMV/exosome preparations (10 μg protein) were lysed in SDS sample buffer, resolved by SDS-PAGE, electrotransferred as previously described^11^,^79^. Membranes were probed with primary mouse anti-CD9 (1:1000, BD Biosciences), mouse anti-Alix (1:1000, Cell Signaling), and mouse anti-A33 (1 μg/ml, a kind gift from Dr. A. Scott, Ludwig, Austin Campus). Membranes were further incubated with secondary antibodies horse radish peroxidase (HRP)-conjugated anti-mouse IgG (1: 15,000, Sigma) and IRDye 800 goat anti-mouse IgG (1: 15000, Li-COR Biosciences). All antibody incubations were carried out using gentle orbital shaking at RT. Proteins were visualised by incubating membranes with Western HRP substrate (Merck-Millipore) followed by imaging with ChemiDoc MP System (Bio-Rad) or imaged directly with the Odyssey Infrared Imaging System, version 3.0 (LI-COR Biosciences, Nebraska USA).

### RNA isolation, library construction and sequencing

Total RNA was extracted using TRIzol^®^ Reagent (Life Technologies) according to the manufacturer’s protocol, as described previously^11^ and the quality and quantity determined using an Agilent 2100 Bioanalyzer (Agilent Technologies). These RNA samples were subsequently used for cDNA library construction (LIM1863 whole cells (CL), A33-Exos, EpCAM-Exos, sMVs) and Illumina sequencing which was performed by Beijing Genomics Institute (BGI, Shenzhen, China). A total amount of 1 μg RNA per sample was used for cDNA library construction using the TruSeq™ RNA Library Preparation Kit v2 protocol (Illumina). Briefly, after DNase digestion and RNA purification, poly(A) mRNA was purified from total RNA using Dynal™ oligo(dT)-attached magnetic beads (Invitrogen-GIBCO). The mRNA was chemically cleaved into small fragments (~200 nt) by exposure to divalent cations under elevated temperature (94 °C, 8 min) in Elute/Prime/Fragment Mix buffer (Illumina). These cleaved RNA fragments were used to synthesize first strand cDNAs using random hexamer-primers and reverse transcriptase (SuperScript^®^ II Reverse Transcriptase, Invitrogen-GIBCO). Second-strand cDNA synthesis was performed using DNA Polymerase I (Invitrogen-GIBCO) and RNase H (Invitrogen-GIBCO). Double-stranded cDNA fragments were purified using AMPure XP beads (Beckman Coulter) and remaining overhangs converted into blunt ends via treatment with T4 DNA polymerase/Klenow DNA polymerase/T4 exonuclease/polymerase using End Repair Mix (Illumina). After adenylation of 3′ ends of DNA fragments using Klenow DNA polymerase the sample was purified using AMPure XP beads. After ligation of Illumina sequencing adaptors to the cDNAs, cDNA fragments were gel-purified using a 1.5% Tris-borate-EDTA polyacrylamide gel (Invitrogen), size-selected (250–350 bp) and amplified by PCR. Amplified cDNA libraries were quality controlled (Agilent 2100 Bioanalyzer and qRT-PCR). The final product should be a band at approximately 260 bp (for single-read libraries). qRT-PCR was used for quantification of sequencing adapter ligation and cDNA libraries were sequenced using an Illumina HiSeq2000 sequencer (typical read lengths 90 bp) and 200 bp paired-end reads were generated.

### Analysis of sequencing results: Mapping and differential expression

Nucleotide sequences were represented based on an image format, where images generated by the sequencer were converted into nucleotide sequences using a base-calling pipeline (Illumina). Raw reads were saved as fastq formatted files. The raw reads were cleaned by removing adaptor sequences, low quality reads containing >50% bases with quality [QA] ≤15, and reads with >2% undefined nucleotides [N]. Clean reads can be publically accessed from the Sequence Read Archive (SRA) of NCBI using the accession number SRA180512. Clean reads were aligned to the human genome sequence and mRNA and ncRNA profiles for each library were determined using the programs TopHat2 and Cufflinks (v2.2.1)^13^,^81^, respectively. Briefly, human genome (GRCh37.74) and gene annotation data were downloaded from Ensembl database (http://www.ensembl.org/index.html) and TopHat2 was used to align the clean reads to the human genome by incorporating the Bowtie2 (http://bowtie-bio.sourceforge.net/bowtie2/index.shtml) algorithm. The processed alignment result file was used to profile mRNA and ncRNA expression using Cufflinks. Gene biotype annotation by Ensembl Automatic Gene Annotation System^14^ and GENCODE^15^ were used to distinguish mRNAs from ncRNAs (pseudogene transcripts, long noncoding RNAs and short noncoding RNAs). Paired alignment reads were used to calculate FPKM (fragments per kilobase of transcript per million mapped reads) values for mRNA/ncRNA transcript expression levels. FKPM values were calculated as follows:


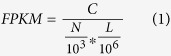


Here C, N and L represent the substitute reads with fragments, the total number of sequenced reads and the length of a particular mRNA/ncRNA, respectively. A cut-off value of >1 FPKM was used as a limit for mRNA/ncRNA detection across all samples. Canonical and variant human mRNAs were classified by linking UniProt and Ensembl annotated protein sequence databases.

mRNAs and ncRNAs enriched in EV samples, relative to CL, were selected using the following criteria: FC (fold change) >2, p-value < 0.05 and a probability >0.7; where FC values represent RNA enrichment changes according to the formula:


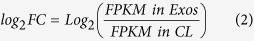


p-values were calculated using the PERL module COMPARISON based on Poisson distribution^82^, and probability values were obtained using the R package NOIseq^83^.

### Identification of novel splicing sites and gene fusions

TopHat2^81^ and ChimeraScan^84^ (v0.4.5) were used to identify alternative splicing and gene fusion events, according to the protocols, respectively.

### Gene ontology analysis

Gene ontology terms for mRNAs enriched in EVs and KEGG pathway analysis were determined using the DAVID Bioinformatics Resource v 6.7 (http://david.abcc.ncifcrf.gov)^85^.

### Correlation of EV-enriched RNAs with gene expression in human cancer patients

EV-enriched mRNAs and lncRNAs identified in this study (relative to CL) were analysed against gene expression data in matched tumour and normal tissues of colon cancer patients from SRA database (https://www.ncbi.nlm.nih.gov/Traces/sra/sra.cgi?). Two SRA studies with accession numbers of SRP022054^76^ and SRP029880^77^ used deep sequencing technology to identify gene expression levels in 4 and 23 pairs of normal/tumour tissues, respectively. Raw sequencing data was obtained and re-analysed based on the criteria established previously for identification of over-expressed mRNAs and lncRNAs.

### Validation of mRNA transcripts using qRT-PCR

Total RNA (CL and EVs) were reverse transcribed into cDNA and synthesised with Reverse Transcription (RT) Master Mix (Applied Biosystems) according to the manufacturer’s instructions. Forward and reverse primers for seven selected mRNAs (SCARB1, SCD, TPT1, EEF1G, BCL7C, RPS3, and RAB13) and three internal controls (CKS1B, GAPDH and MLF2) were designed using OligoArchitec^TM^ Online (Sigma-Aldrich, for primer sequences, see Supplementary Table 7) and synthesised by Integrated DNA Technology Inc. PCR experiments were optimised to determine the sample concentration, primer concentration and reaction temperature for each primer pair. SsoAdvanced^TM^ Universal Supermixes (5 μl, Bio-Rad Laboratories Inc.), forward primer (10 μM, 0.5 μl), reverse primer (10 μM, 0.5 μl) and RNase-free H_2_O (2 μl) were added to 2 μl of cDNA sample (12 ng/μl) per reaction. Technical triplicate analyses for each mRNA were performed for each sample on CFX96 Touch^TM^ Real-Time PCR Detection System (Bio-Rad Laboratories Inc.). The CFX Manager^TM^ Software (v3.1) was used to analyse the mRNA expression levels and to calculate the expression changes between samples.

## Additional Information

**How to cite this article**: Chen, M. *et al*. Transcriptome and long noncoding RNA sequencing of three extracellular vesicle subtypes released from the human colon cancer LIM1863 cell line. *Sci. Rep.* **6**, 38397; doi: 10.1038/srep38397 (2016).

**Publisher's note:** Springer Nature remains neutral with regard to jurisdictional claims in published maps and institutional affiliations.

## Supplementary Material

Supplementary Information

Supplementary Dataset

## Figures and Tables

**Table 1:** Number of snRNAs selectively distributed into LIM1863 EV subtypes.

snRNA	sMVs (35)	A33-Exos (23)	EpCAM-Exos (49)
U1	8	5	9
Variant U1*	5	3	6
U2	0	0	3
U4	2	3	4
U5	1	0	0
U6	18	11	26
U6atac	1	1	1

^*^Variant U1 snRNAs, from different gene locuses.

**Table 2:** snoRNAs from different families enriched in sMVs, A33-Exos and EpCAM-Exos.

Family	sMVs (11)	A33-Exos (19)	EpCAM-Exos (14)
H/ACA box snoRNA	SNORA25, SNORA62, SNORA71A, NORA71C, SNORA72, SNORA76, snoU109	SNORA18, SNORA27, SNORA57, SNORA62, SNORA68, SNORA70, SNORA77	SNORA14B, SNORA31, SNORA76, SNORA77
C/D box snoRNA	SNORD94, U3, snoU13	SNORD23, snoU13	U3, snoU13
scaRNA	—	—	SCARNA16, SCARNA22
Other	SNHG12	SNHG12	—

**Table 3:** RNA binding proteins identified in this study and their RNA binding partners.

RBP^a^	sMVs				A33-Exos				EpCAM-Exos			
	mRNA^b^	pseudogene^c^	lncRNA^d^	sncRNA^e^	mRNA^b^	pseudogene^c^	lncRNA^d^	sncRNA^e^	mRNA^b^	pseudogene^c^	lncRNA^d^	sncRNA^e^
SFRS1	788	110	27	2	—	—	—	—	1112	145	37	1
eIF4AIII	1098	8	43	11	—	—	—	—	1602	9	85	21
UPF1	1063	62	37	20	—	—	—	—	—	—	—	—
PTB	702	8	16	7	981	10	28	23	1078	6	32	25
hnRNPC	510	5	25	7	—	—	—	—	747	4	44	23
U2AF65	1021	25	35	9	—	—	—	—	—	—	—	—
TAF15	255	1	22	1	—	—	—	-	—	—	—	—

^a^RNA binding proteins identified in this study using MS data.

^b^EV-enriched mRNAs which can bind to the RBP.

^c^EV-enriched pseudogene transcripts which can bind to the RBP.

^d^EV-enriched long noncoding RNAs which can bind to the RBP.

^e^EV-enriched short noncoding RNAs which can bind to the RBP.

**Table 4:** EV-enriched mRNAs and lncRNAs in CRC and other cancers.

Gene	Cancer type	Samples	Regulation (tumour vs normal)	RNA/protein	Reference
EV-enriched mRNAs:					
TPT1	CRC	Tumour washing fluid.	Up	protein	PMID: 22294321
	CRC and bladder cancer	28 tumor biopsy samples from bladder cancer, 10 normal and 30 tumor from colon cancer.	Up	RNA	PMID: 15289330
	CRC	Stool samples were obtained from 23 patients with colorectal cancer (Dukes stages A-C) before surgical resection and 15 healthy volunteers a few weeks after they had undergone a total colonoscopy.	Up	RNA	PMID: 17912428
	CRC	16 matched normal and tumor tissues from CRC patients; 109 serum samples of CRC patients.	Up	protein	PMID: 16166432
	Oral cancer	Plasma from primary oral squamous cell carcinoma (OSCC) and their matched adjacent normal surrounding mucosa specimens from 20 patients.	Up	RNA/protein	PMID: 22902387
	Oral cancer	Blood samples were collected from patients (n = 32) with primary T1/T2 OSCC and matched healthy patients (n = 35).	Up	RNA	PMID: 16505414
RPS3, S6, S8, S12, L5, and P0	CRC	Tumors and polyps	Up	RNA	PMID: 1712897
Ribosome-associated (RPL17; RPL22; RPL35A; RPL37; RPL41; RPLP1; RPS13; RPS23; RPS3; RPS3A; UBA52)	CRC	82 pairs of CRC tumor tissues and corresponding adjacent non-cancerous tissues.	Down	RNA	PMID: 16773188
48 RP genes (no detail)	CRC	Normal/tumor tissue; cell line.	Up	RNA	PMID: 9157888
RPL8, L18, L18a, L29, L6, L3, S19, L7, S5	CRC	Matched tumor and corresponding normal mucosae tissues from 20 CRC patients.	Up	RNA	PMID: 11325815
RPS3, S4X, S27a, S3, L6, L9, S3A, S2, L3	CRC	Eleven differentiated adenocarcinomas, nine adenomas, and their corresponding normal mucosae of the colon.	Up	RNA	PMID: 12037668
EEF1A1	CRC	45 tissue samples and five cell lines.	Up	RNA	PMID: 14973550
	CRC	HCT-116 (wt-p53) and HCT-116 (null-p53).	Up	RNA	PMID: 16609010
EEF1B2	CRC	Two colon biopsies of tubular adenomas with focal malignant changes.	Up	RNA	PMID: 16969489
FTL	CRC	Stool samples were obtained from 23 patients with colorectal cancer (Dukes stages A-C) before surgical resection and 15 healthy volunteers a few weeks after they had undergone a total colonoscopy.	Up	RNA	PMID: 17912428
EV-enriched lncRNAs:					
SNHG5	Gastric cancer	Paired gastric cancer and corresponding non-tumor tissues of 10 patients (cohort 1); 23 normal gastric epithelial tissues (cohort 2) and 87 paired tumor and non-tumor gastric specimens from patients with GC (cohort 3).	Down	RNA	PMID: 27065326
SNHG6	Hepatocellular Carcinoma	A total of 360 patients with HCC were retrieved from the TCGA data portal.	Up	RNA	PMID: 26492393
SNHG7			Up	RNA	PMID: 26492393
GAS5	Hepatocellular carcinoma	A total of 71 paired clinical HCC tissues and adjacent normal tissues	Down	RNA	PMID: 25120813
	CRC	Tumor tissues and corresponding non-tumor colorectal tissues from 66 CRC patients.	Down	RNA	PMID: 25326054
	Pancreatic cancer	23 specimens of pancreatic cancer tissue, 10 samples of normal pancreas following trauma; Human pancreatic cancer cells (BxPC-3, PANC-1, AsPC-1, and Hs766T).	Down	RNA	PMID: 24026436
ZFAS1	CRC	119 paired tumor and normal colorectal tissues.	Up	RNA	PMID: 26506418
H19	Gastric cancer	22 specimens of gastric cancer tissues and adjacent benign tissues.	Up	RNA	PMID: 22776265
	CRC	Human CRC cell lines: 228, CaCO2, Clone A, HCT116, HT-29, MIP101, SW480; Normal colon fibroblast cell lines: CCD-112CoN, CCD-18Co; 30 matched primary CRC and their adjacent non-cancerous tissues.	Up	RNA	PMID: 19926638
LINC00116	Ovarian endometriosis	Ectopic endometrium (EC) and paired eutopic endometrium (EU) tissues were obtained from the cyst wall of 25 women with ovarian endometriosis during laparoscopic surgeries in the period of October 2012 to March 2013.	Up	RNA	PMID: 24502888
EPB41L4A-AS1	Pancreatic cancer	Pancreatic cancer tissue from 6 patients and normal pancreatic tissue from 5 controls.	Up	RNA	PMID: 25910082
MAPKAPK5-AS1	Hepatocellular Carcinoma	A total of 360 patients with HCC were retrieved from the TCGA data portal.	Up	RNA	PMID: 26492393

## References

[b1] Xu R., Greening D. W., Zhu H. J., Takahashi N. & Simpson R. J. Extracellular vesicle isolation and characterization: toward clinical application. J Clin Invest 126, 1152–1162 (2016). 27035807 10.1172/JCI81129 PMC4811150

[b2] Al-Nedawi K. . Intercellular transfer of the oncogenic receptor EGFRvIII by microvesicles derived from tumour cells. Nat Cell Biol 10, 619–624 (2008). 18425114 10.1038/ncb1725

[b3] Di Vizio D. . Large oncosomes in human prostate cancer tissues and in the circulation of mice with metastatic disease. Am J Pathol 181, 1573–1584 (2012). 23022210 10.1016/j.ajpath.2012.07.030 PMC3483805

[b4] Ma L. . Discovery of the migrasome, an organelle mediating release of cytoplasmic contents during cell migration. Cell Res 25, 24–38 (2015). 25342562 10.1038/cr.2014.135 PMC4650581

[b5] Mathivanan S. . Proteomics analysis of A33 immunoaffinity-purified exosomes released from the human colon tumor cell line LIM1215 reveals a tissue-specific protein signature. Mol Cell Proteomics 90 9, 197–208 (2010). 10.1074/mcp.M900152-MCP200 PMC2830834 19837982

[b6] Ji H. . Proteome profiling of exosomes derived from human primary and metastatic colorectal cancer cells reveal differential expression of key metastatic factors and signal transduction components. Proteomics 13, 1672–1686 (2013). 23585443 10.1002/pmic.201200562

[b7] Tauro B. J. . Comparison of ultracentrifugation, density gradient separation, and immunoaffinity capture methods for isolating human colon cancer cell line LIM1863-derived exosomes. Methods 56, 293–304 (2012). 22285593 10.1016/j.ymeth.2012.01.002

[b8] Tauro B. J. . Two distinct populations of exosomes are released from LIM1863 colon carcinoma cell-derived organoids. Mol Cell Proteomics s 12, 587–598 (2013). 10.1074/mcp.M112.021303 PMC3591653 23230278

[b9] Glotzer M. The 3Ms of central spindle assembly: microtubules, motors and MAPs. Nat Rev Mol Cell Biol 10, 9–20 (2009). 19197328 10.1038/nrm2609 PMC2789570

[b10] Xu R., Greening D. W., Rai A., Ji H. & Simpson R. J. Highly-purified exosomes and shed microvesicles isolated from the human colon cancer cell line LIM1863 by sequential centrifugal ultrafiltration are biochemically and functionally distinct. Methods 87, 11–25 (2015). 25890246 10.1016/j.ymeth.2015.04.008

[b11] Ji H. . Deep sequencing of RNA from three different extracellular vesicle (EV) subtypes released from the human LIM1863 colon cancer cell line uncovers distinct miRNA-enrichment signatures. PLoS One 9, e110314 (2014). 25330373 10.1371/journal.pone.0110314 PMC4201526

[b12] S E. L. A., Mager I., Breakefield X. O. & Wood M. J. Extracellular vesicles: biology and emerging therapeutic opportunities. Nat Rev Drug Discov 12, 347–357 (2013). 23584393 10.1038/nrd3978

[b13] Trapnell C. . Differential gene and transcript expression analysis of RNA-seq experiments with TopHat and Cufflinks. Nat Protoc 7, 562–578 (2012). 22383036 10.1038/nprot.2012.016 PMC3334321

[b14] Curwen V. . The Ensembl automatic gene annotation system. Genome Res 14, 942–950 (2004). 15123590 10.1101/gr.1858004 PMC479124

[b15] Harrow J. . GENCODE: the reference human genome annotation for The ENCODE Project. Genome Res 22, 1760–1774 (2012). 22955987 10.1101/gr.135350.111 PMC3431492

[b16] Chang Y. F., Imam J. S. & Wilkinson M. F. The nonsense-mediated decay RNA surveillance pathway. Annu Rev Biochem 76, 51–74 (2007). 17352659 10.1146/annurev.biochem.76.050106.093909

[b17] Vasudevan S., Peltz S. W. & Wilusz C. J. Non-stop decay–a new mRNA surveillance pathway. Bioessays 24, 785–788 (2002). 12210514 10.1002/bies.10153

[b18] Cunningham F. . Ensembl 2015. Nucleic Acids Res 43, D662–669 (2015). 25352552 10.1093/nar/gku1010 PMC4383879

[b19] Yates A. . Ensembl 2016. Nucleic Acids Res 44, D710–716 (2016). 26687719 10.1093/nar/gkv1157 PMC4702834

[b20] O’Donovan C. & Apweiler R. A guide to UniProt for protein scientists. Methods Mol Biol 694, 25–35 (2011). 21082425 10.1007/978-1-60761-977-2_2

[b21] UniProt C. The Universal Protein Resource (UniProt) in 2010. Nucleic Acids Res 38, D142–148 (2010). 19843607 10.1093/nar/gkp846 PMC2808944

[b22] UniProt C. Reorganizing the protein space at the Universal Protein Resource (UniProt). Nucleic Acids Res 40, D71–75 (2012). 22102590 10.1093/nar/gkr981 PMC3245120

[b23] Silvera D., Formenti S. C. & Schneider R. J. Translational control in cancer. Nat Rev Cancer 10, 254–266 (2010). 20332778 10.1038/nrc2824

[b24] Grzmil M. & Hemmings B. A. Translation regulation as a therapeutic target in cancer. Cancer Res 72, 3891–3900 (2012). 22850420 10.1158/0008-5472.CAN-12-0026

[b25] Huang da W., Sherman B. T. & Lempicki R. A. Systematic and integrative analysis of large gene lists using DAVID bioinformatics resources. Nat Protoc 4, 44–57 (2009). 19131956 10.1038/nprot.2008.211

[b26] Huang da W., Sherman B. T. & Lempicki R. A. Bioinformatics enrichment tools: paths toward the comprehensive functional analysis of large gene lists. Nucleic Acids Res 37, 1–13 (2009). 19033363 10.1093/nar/gkn923 PMC2615629

[b27] Breuza L. . The UniProtKB guide to the human proteome. Database 2016 (2016). 10.1093/database/bav120 PMC4761109 26896845

[b28] Kim M. S. . A draft map of the human proteome. Nature 509, 575–581 (2014). 24870542 10.1038/nature13302 PMC4403737

[b29] Wilhelm M. . Mass-spectrometry-based draft of the human proteome. Nature 509, 582–587 (2014). 24870543 10.1038/nature13319

[b30] San Lucas F. A. . Minimally invasive genomic and transcriptomic profiling of visceral cancers by next-generation sequencing of circulating exosomes. Ann Oncol 27, 635–641 (2016). 26681674 10.1093/annonc/mdv604 PMC4803451

[b31] Schumacher T. N. & Schreiber R. D. Neoantigens in cancer immunotherapy. Science 348, 69–74 (2015). 25838375 10.1126/science.aaa4971

[b32] Lasho T. . Identification of submicroscopic genetic changes and precise breakpoint mapping in myelofibrosis using high resolution mate-pair sequencing. Am J Hematol 88, 741–746 (2013). 23733509 10.1002/ajh.23495

[b33] Mehra R. . Primary urethral clear-cell adenocarcinoma: comprehensive analysis by surgical pathology, cytopathology, and next-generation sequencing. Am J Pathol 184, 584–591 (2014). 24389164 10.1016/j.ajpath.2013.11.023 PMC3936309

[b34] Kekeeva T. . Novel fusion transcripts in bladder cancer identified by RNA-seq. Cancer Lett 374, 224–228 (2016). 26898937 10.1016/j.canlet.2016.02.010

[b35] Baker N. E. & Kale A. Mutations in ribosomal proteins: Apoptosis, cell competition, and cancer. Mol Cell Oncol 3, e1029065 (2016). 27308545 10.1080/23723556.2015.1029065 PMC4845181

[b36] Meng X. . RPL23 links oncogenic RAS signaling to p53-mediated tumor suppression. Cancer Res 76, 5030–5039 (2016). 27402081 10.1158/0008-5472.CAN-15-3420 PMC5693225

[b37] Rao S. . Ribosomal Protein Rpl22 Controls the Dissemination of T-cell Lymphoma. Cancer Res 76, 3387–3396 (2016). 27197189 10.1158/0008-5472.CAN-15-2698 PMC4891229

[b38] Poliseno L. Pseudogenes: newly discovered players in human cancer. Sci Signal 5, re5 (2012). 22990117 10.1126/scisignal.2002858

[b39] Pink R. C. . Pseudogenes: pseudo-functional or key regulators in health and disease? RNA 17, 792–798 (2011). 21398401 10.1261/rna.2658311 PMC3078729

[b40] Consortium E. P. . Identification and analysis of functional elements in 1% of the human genome by the ENCODE pilot project. Nature 447, 799–816 (2007). 17571346 10.1038/nature05874 PMC2212820

[b41] Mercer T. R., Dinger M. E. & Mattick J. S. Long non-coding RNAs: insights into functions. Nat Rev Genet 10, 155–159 (2009). 19188922 10.1038/nrg2521

[b42] Qiu M. T., Hu J. W., Yin R. & Xu L. Long noncoding RNA: an emerging paradigm of cancer research. Tumour Biol 34, 613–620 (2013). 23359273 10.1007/s13277-013-0658-6

[b43] Tsang W. P. . Oncofetal H19-derived miR-675 regulates tumor suppressor RB in human colorectal cancer. Carcinogenesis 31, 350–358 (2010). 19926638 10.1093/carcin/bgp181

[b44] Walter P. & Blobel G. Signal recognition particle contains a 7S RNA essential for protein translocation across the endoplasmic reticulum. Nature 299, 691–698 (1982). 6181418 10.1038/299691a0

[b45] Zwieb C., van Nues R. W., Rosenblad M. A., Brown J. D. & Samuelsson T. A nomenclature for all signal recognition particle RNAs. RNA 11, 7–13 (2005). 15611297 10.1261/rna.7203605 PMC1370686

[b46] Lerner M. R., Boyle J. A., Hardin J. A. & Steitz J. A. Two novel classes of small ribonucleoproteins detected by antibodies associated with lupus erythematosus. Science 211, 400–402 (1981). 6164096 10.1126/science.6164096

[b47] Christov C. P., Gardiner T. J., Szuts D. & Krude T. Functional requirement of noncoding Y RNAs for human chromosomal DNA replication. Mol Cell Biol 26, 6993–7004 (2006). 16943439 10.1128/MCB.01060-06 PMC1592862

[b48] Zhang A. T. . Dynamic interaction of Y RNAs with chromatin and initiation proteins during human DNA replication. J Cell Sci 124, 2058–2069 (2011). 21610089 10.1242/jcs.086561 PMC3104036

[b49] Lai R. C. . MSC secretes at least 3 EV types each with a unique permutation of membrane lipid, protein and RNA. J Extracell Vesicles 5, 29828 (2016). 26928672 10.3402/jev.v5.29828 PMC4770866

[b50] Matera A. G. & Wang Z. A day in the life of the spliceosome. Nat Rev Mol Cell Biol 15, 108–121 (2014). 24452469 10.1038/nrm3742 PMC4060434

[b51] Jorjani H. . An updated human snoRNAome. Nucleic Acids Res (2016). 10.1093/nar/gkw386 PMC4914119 27174936

[b52] Krogh N. . Profiling of 2’-O-Me in human rRNA reveals a subset of fractionally modified positions and provides evidence for ribosome heterogeneity. Nucleic Acids Res (2016). 10.1093/nar/gkw482 PMC5027482 27257078

[b53] Ponting C. P., Oliver P. L. & Reik W. Evolution and functions of long noncoding RNAs. Cell 136, 629–641 (2009). 19239885 10.1016/j.cell.2009.02.006

[b54] Paquet E. R. . A 12-gene signature to distinguish colon cancer patients with better clinical outcome following treatment with 5-fluorouracil or FOLFIRI. J Path Clin Res 1, 160–172 (2015). 27499901 10.1002/cjp2.17 PMC4939880

[b55] Gerbasi F. R., Bottoms S., Farag A. & Mammen E. F. Changes in hemostasis activity during delivery and the immediate postpartum period. Am J Obstet Gynecol 162, 1158–1163 (1990). 2140236 10.1016/0002-9378(90)90006-s

[b56] Li T. . Amplification of Long Noncoding RNA ZFAS1 Promotes Metastasis in Hepatocellular Carcinoma. Cancer Res 75, 3181–3191 (2015). 26069248 10.1158/0008-5472.CAN-14-3721

[b57] Thorenoor N. . Long non-coding RNA ZFAS1 interacts with CDK1 and is involved in p53-dependent cell cycle control and apoptosis in colorectal cancer. Oncotarget 7, 622–637 (2016). 26506418 10.18632/oncotarget.5807 PMC4808022

[b58] Mourtada-Maarabouni M., Pickard M. R., Hedge V. L., Farzaneh F. & Williams G. T. GAS5, a non-protein-coding RNA, controls apoptosis and is downregulated in breast cancer. Oncogene 28, 195–208 (2009). 18836484 10.1038/onc.2008.373

[b59] Pickard M. R., Mourtada-Maarabouni M. & Williams G. T. Long non-coding RNA GAS5 regulates apoptosis in prostate cancer cell lines. Biochim Biophys Acta 1832, 1613–1623 (2013). 23676682 10.1016/j.bbadis.2013.05.005

[b60] Sun M. . Decreased expression of long noncoding RNA GAS5 indicates a poor prognosis and promotes cell proliferation in gastric cancer. BMC Cancer 14, 319 (2014). 24884417 10.1186/1471-2407-14-319 PMC4022532

[b61] Shi X. . A critical role for the long non-coding RNA GAS5 in proliferation and apoptosis in non-small-cell lung cancer. Mol Carcinog 54 **Suppl 1**, E1–E12 (2015). 24357161 10.1002/mc.22120

[b62] Liu Z. . Downregulation of GAS5 promotes bladder cancer cell proliferation, partly by regulating CDK6. PLoS One 8, e73991 (2013). 24069260 10.1371/journal.pone.0073991 PMC3775789

[b63] Yu H. . Identification and validation of long noncoding RNA biomarkers in human non-small-cell lung carcinomas. J Thorac Oncol 10, 645–654 (2015). 25590602 10.1097/JTO.0000000000000470

[b64] Rinn J. L. & Chang H. Y. Genome regulation by long noncoding RNAs. Annu Rev Biochem 81, 145–166 (2012). 22663078 10.1146/annurev-biochem-051410-092902 PMC3858397

[b65] Gerstberger S., Hafner M. & Tuschl T. A census of human RNA-binding proteins. Nat Rev Genet 15, 829–845 (2014). 25365966 10.1038/nrg3813 PMC11148870

[b66] Tauro B. J. . Two distinct population of exosomes released from LIM1863 colon caricinoma cells. Mol Cell Proteomics 12, 587–598 (2013). 23230278 10.1074/mcp.M112.021303 PMC3591653

[b67] Cook K. B., Kazan H., Zuberi K., Morris Q. & Hughes T. R. RBPDB: a database of RNA-binding specificities. Nucleic Acids Res 39, D301–308 (2011). 21036867 10.1093/nar/gkq1069 PMC3013675

[b68] Han S. P., Tang Y. H. & Smith R. Functional diversity of the hnRNPs: past, present and perspectives. Biochem J 430, 379–392 (2010). 20795951 10.1042/BJ20100396

[b69] Fairman-Williams M. E., Guenther U. P. & Jankowsky E. SF1 and SF2 helicases: family matters. Curr Opin Struct Biol. 20, 313–324 (2010). 20456941 10.1016/j.sbi.2010.03.011 PMC2916977

[b70] Villarroya-Beltri C. . Sumoylated hnRNPA2B1 controls the sorting of miRNAs into exosomes through binding to specific motifs. Nat Commun 4, 2980 (2013). 24356509 10.1038/ncomms3980 PMC3905700

[b71] Li J. H., Liu S., Zhou H., Qu L. H. & Yang J. H. StarBase v2.0: decoding miRNA-ceRNA, miRNA-ncRNA and protein-RNA interaction networks from large-scale CLIP-Seq data. Nucleic Acids Res 42, D92–97 (2014). 24297251 10.1093/nar/gkt1248 PMC3964941

[b72] Yin D. . Long noncoding RNA GAS5 affects cell proliferation and predicts a poor prognosis in patients with colorectal cancer. Med Oncol 31, 253 (2014). 25326054 10.1007/s12032-014-0253-8

[b73] Tu Z. Q., Li R. J., Mei J. Z. & Li X. H. Down-regulation of long non-coding RNA GAS5 is associated with the prognosis of hepatocellular carcinoma. Int J Clin Exp Pathol 7, 4303–4309 (2014). 25120813 PMC4129048

[b74] Lu X. . Downregulation of gas5 increases pancreatic cancer cell proliferation by regulating CDK6. Cell Tissue Res 354, 891–896 (2013). 24026436 10.1007/s00441-013-1711-x

[b75] Putz U. . The tumor suppressor PTEN is exported in exosomes and has phosphatase activity in recipient cells. Sci Signal 5, ra70 (2012). 23012657 10.1126/scisignal.2003084

[b76] Rohr C. . High-throughput miRNA and mRNA sequencing of paired colorectal normal, tumor and metastasis tissues and bioinformatic modeling of miRNA-1 therapeutic applications. PLoS One 8, e67461 (2013). 23874421 10.1371/journal.pone.0067461 PMC3707605

[b77] Kim S. K. . A nineteen gene-based risk score classifier predicts prognosis of colorectal cancer patients. Molecular oncology 8, 1653–1666 (2014). 25049118 10.1016/j.molonc.2014.06.016 PMC5528589

[b78] Whitehead R. H., Jones J. K., Gabriel A. & Lukies R. E. A new colon carcinoma cell line (LIM1863) that grows as organoids with spontaneous differentiation into crypt-like structures *in vitro*. Cancer Research 47, 2683–2689 (1987). 3567898

[b79] Greening D. W., Xu R., Ji H., Tauro B. J. & Simpson R. J. A protocol for exosome isolation and characterization: evaluation of ultracentrifugation, density-gradient separation, and immunoaffinity capture methods. Meth Mol Biol 1295, 179–209 (2015). 10.1007/978-1-4939-2550-6_15 25820723

[b80] Tauro B. J. . Oncogenic H-ras reprograms Madin-Darby canine kidney (MDCK) cell-derived exosomal proteins following epithelial-mesenchymal transition. Mol Cell Proteomics 12, 2148–2159 (2013). 23645497 10.1074/mcp.M112.027086 PMC3734576

[b81] Kim D. . TopHat2: accurate alignment of transcriptomes in the presence of insertions, deletions and gene fusions. Genome Biol 14, R36 (2013). 23618408 10.1186/gb-2013-14-4-r36 PMC4053844

[b82] Audic S. & Claverie J. M. The significance of digital gene expression profiles. Genome Res 7, 986–995 (1997). 9331369 10.1101/gr.7.10.986

[b83] Tarazona S., García F., Ferrer A., Dopazo J. & Conesa A. NOIseq: a RNA-seq differential expression method robust for sequencing depth biases. EMBnet. 17, 18–19 (2012).

[b84] Iyer M. K., Chinnaiyan A. M. & Maher C. A. ChimeraScan: a tool for identifying chimeric transcription in sequencing data. Bioinformatics 27, 2903–2904 (2011). 21840877 10.1093/bioinformatics/btr467 PMC3187648

[b85] Huang D. W. . DAVID Bioinformatics Resources: expanded annotation database and novel algorithms to better extract biology from large gene lists. Nucleic Acids Res 35, W169–175 (2007). 17576678 10.1093/nar/gkm415 PMC1933169

[b86] Kondo Y., Oubridge C., van Roon A. M. & Nagai K. Crystal structure of human U1 snRNP, a small nuclear ribonucleoprotein particle, reveals the mechanism of 5’ splice site recognition. Elife 4 (2015). 10.7554/eLife.04986 PMC4383343 25555158

[b87] Kuwasako K. . Solution structures of the SURP domains and the subunit-assembly mechanism within the splicing factor SF3a complex in 17S U2 snRNP. Structure 14, 1677–1689 (2006). 17098193 10.1016/j.str.2006.09.009

