# Supplementary material for: Transcriptome and long noncoding RNA sequencing of three extracellular vesicle subtypes released from the human colon cancer LIM1863 cell line
Source: Sci Rep. 2016 Dec 5;6:38397. doi: 10.1038/srep38397 (PMC5137021; doi:10.1038/srep38397)
Supplement: Supplementary Information [file srep38397-s1.pdf]

Supplementary Information

Transcriptome and long noncoding RNA sequencing of three extracellular vesicle subtypes released from the human colon cancer LIM1863 cell line

Maoshan Chen[1], Rong Xu[1], Hong Ji[1], David W. Greening[1], Alin Rai[1], Keiichi Izumikawa[2,3], Hideaki Ishikawa[2,3], Nobuhiro Takahashi[2,3] and Richard J. Simpson[1,3]*

[1]Department of Biochemistry and Genetics, La Trobe Institute for Molecular Science (LIMS), La Trobe University, Melbourne, Victoria, Australia

[2]Department of Applied Biological Science, Graduate School of Agriculture, Tokyo University of Agriculture and Technology, Tokyo, Japan

[3]Global Innovation Research Organisation, Tokyo University of Agriculture and Technology, Tokyo, Japan

*__Correspondence to__:

Professor Richard J. Simpson PhD FATSE

Department of Biochemistry and Genetics

La Trobe Institute for Molecular Science (LIMS)

La Trobe University, Bundoora Victoria 3086

Australia

Tel: +61 3 9479 3099

EM: Richard.simpson@latrobe.edu.au

Keywords: Exosomes, colon cancer, RNA-Seq, lncRNA, mRNA, LIM1863, RNA binding proteins, Ribonucleoproteins, shed microvesicles, microparticles

Supplementary Figures

Fig. S1

A

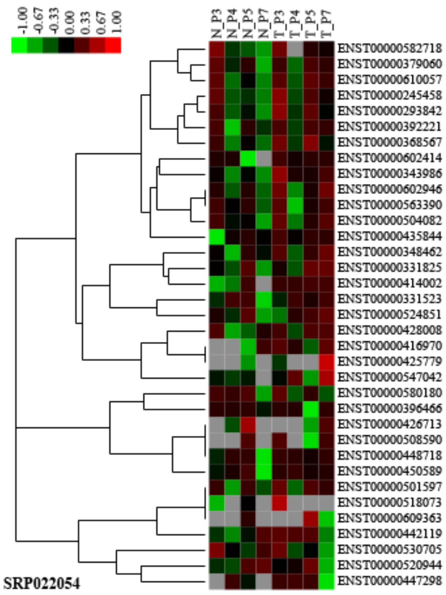

B

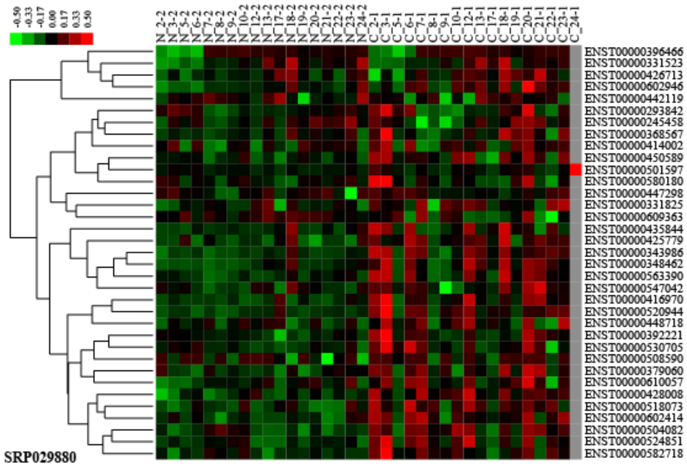

Supplementary Tables

Table S1. Significant KEGG pathway for mRNAs encoding canonical proteins commonly and specifically enriched in sMVs, A33-Exos and EpCAM-Exos.

RNA source	Term	Count	%	PValue	-LOG10(FDR)	Fold Enrichment	Benjamini
Common	hsa03010:Ribosome	67	36.02	1.39E-101	100.86	3.53E+01	6.41E-100
	hsa00190:Oxidative phosphorylation	15	8.06	5.76E-07	6.24	5.29E+00	1.33E-05
	hsa05012:Parkinson's disease	13	6.99	1.67E-05	4.78	4.65E+00	2.56E-04
	hsa05010:Alzheimer's disease	13	6.99	1.81E-04	3.74	3.65E+00	2.08E-03
	hsa05016:Huntington's disease	13	6.99	4.55E-04	3.34	3.31E+00	4.18E-03
	hsa04260:Cardiac muscle contraction	6	3.23	2.62E-02	1.58	3.52E+00	1.84E-01
A33-Exos special	hsa03010:Ribosome	12	8.05	2.28E-09	8.64	1.19E+01	1.48E-07
	hsa05012:Parkinson's disease	6	4.03	1.48E-02	1.83	4.04E+00	3.83E-01
	hsa05016:Huntington's disease	7	4.70	1.59E-02	1.80	3.35E+00	2.94E-01
	hsa00480:Glutathione metabolism	4	2.68	1.89E-02	1.72	6.89E+00	2.67E-01
	hsa00020:Citrate cycle (TCA cycle)	3	2.01	4.81E-02	1.32	8.34E+00	4.73E-01
	hsa00280:Valine, leucine and isoleucine degradation	3	2.01	8.92E-02	1.05	5.88E+00	6.37E-01
	hsa03050:Proteasome	3	2.01	9.98E-02	1.00	5.50E+00	6.23E-01
EpCAM-Exos special	hsa03010:Ribosome	11	5.00	1.40E-06	5.86	7.48E+00	1.47E-04
	hsa03040:Spliceosome	12	5.45	6.29E-06	5.20	5.63E+00	3.30E-04
	hsa05016:Huntington's disease	13	5.91	3.69E-05	4.43	4.27E+00	1.29E-03
	hsa05012:Parkinson's disease	10	4.55	2.50E-04	3.60	4.62E+00	6.55E-03
	hsa00190:Oxidative phosphorylation	10	4.55	2.82E-04	3.55	4.55E+00	5.90E-03
	hsa05010:Alzheimer's disease	10	4.55	1.46E-03	2.84	3.63E+00	2.52E-02
	hsa04110:Cell cycle	6	2.73	5.72E-02	1.24	2.84E+00	5.87E-01

Table S2. Biological process GO annotation for mRNAs encoding isoform proteins commonly and specifically enriched in sMVs, A33-Exos and EpCAM-Exos.

RNA source	GO_ID	GO_term	Count	%	PValue	-LOG10(FDR)	Fold Enrichment	Benjamini
Common	GO:0006414	translational elongation	5	10.2	1.60E-04	3.80	17.62	5.61E-02
	GO:0006412	translation	7	14.29	2.51E-04	3.6	7.53	4.43E-02
sMVs special	GO:0034622	cellular macromolecular complex assembly	4	25	2.42E-03	2.62	13.09	4.61E-01
	GO:0034621	cellular macromolecular complex subunit organization	4	25	3.36E-03	2.47	11.66	3.49E-01
A33-Exos special	GO:0006091	generation of precursor metabolites and energy	5	9.09	1.12E-02	1.95	5.54	9.78E-01
	GO:0051436	negative regulation of ubiquitin-protein ligase activity during mitotic cell cycle	3	5.45	1.43E-02	1.85	16.01	9.13E-01
	GO:0031145	anaphase-promoting complex-dependent proteasomal ubiquitin-dependent protein catabolic process	3	5.45	1.43E-02	1.85	16.01	9.13E-01
	GO:0051444	negative regulation of ubiquitin-protein ligase activity	3	5.45	1.51E-02	1.82	15.53	8.22E-01
	GO:0051352	negative regulation of ligase activity	3	5.45	1.51E-02	1.82	15.53	8.22E-01
	GO:0051437	positive regulation of ubiquitin-protein ligase activity during mitotic cell cycle	3	5.45	1.56E-02	1.81	15.3	7.36E-01
	GO:0051443	positive regulation of ubiquitin-protein ligase activity	3	5.45	1.65E-02	1.784	14.87	6.75E-01
	GO:0051439	regulation of ubiquitin-protein ligase activity during mitotic cell cycle	3	5.45	1.69E-02	1.772	14.66	6.18E-01
	GO:0006793	phosphorus metabolic process	8	14.55	1.72E-02	1.763	2.85	5.69E-01
	GO:0006796	phosphate metabolic process	8	14.55	1.72E-02	1.763	2.85	5.69E-01
	GO:0051351	positive regulation of ligase activity	3	5.45	1.78E-02	1.755	14.26	5.33E-01
	GO:0031397	negative regulation of protein ubiquitination	3	5.45	1.83E-02	1.74	14.06	5.01E-01
	GO:0051438	regulation of ubiquitin-protein ligase activity	3	5.45	2.02E-02	1.70	13.34	4.99E-01
	GO:0051340	regulation of ligase activity	3	5.45	2.17E-02	1.66	12.85	4.91E-01
	GO:0031398	positive regulation of protein ubiquitination	3	5.45	2.32E-02	1.63	12.39	4.84E-01
	GO:0045333	cellular respiration	3	5.45	3.03E-02	1.52	10.73	5.51E-01
	GO:0006119	oxidative phosphorylation	3	5.45	3.08E-02	1.51	10.62	5.32E-01
EpCAM-Exos special	GO:0009057	macromolecule catabolic process	10	16.95	5.41E-04	3.27	4.03	3.10E-01
	GO:0032446	protein modification by small protein conjugation	5	8.47	7.27E-04	3.14	11.92	2.21E-01
	GO:0070647	protein modification by small protein conjugation or removal	5	8.47	1.49E-03	2.83	9.83	0.288036
	GO:0044265	cellular macromolecule catabolic process	9	15.25	1.52E-03	2.82	3.91	0.2299769
	GO:0030163	protein catabolic process	8	13.56	2.78E-03	2.56	4.05	0.3173619
	GO:0010605	negative regulation of macromolecule metabolic process	8	13.56	6.89E-03	2.16	3.43	0.5457988
	GO:0043632	modification-dependent macromolecule catabolic process	7	11.86	8.12E-03	2.09	3.84	0.5497784
	GO:0019941	modification-dependent protein catabolic process	7	11.86	8.12E-03	2.09	3.84	0.5497784
	GO:0051603	proteolysis involved in cellular protein catabolic process	7	11.86	9.99E-03	2	3.67	0.5767992

Table S3. mRNAs with missing proteins in UniProtKB that cannot find EV-enriched mRNAs encoding canonical/isoform proteins.

tracking_id	gene_name	Expression (FPKM)				sMVs/CL			A33-Exos/CL			EpCAM-Exos/CL		
		CL	sMVs	A33-Exos	EpCAM-Exos	Log2FC	p-value	probability	Log2FC	p-value	probability	Log2FC	p-value	probability
ENST00000256463	BRK1	70.994	88.932	142.771	153.194	0.33	1.57E-01	0.60	1.01	7.68E-07	0.9	1.11	3.03E-08	0.91
ENST00000406213	AP000350.4	26.797	67.986	101.084	67.968	1.34	1.92E-05	0.91	1.92	1.52E-11	0.9	1.34	1.93E-05	0.92
ENST00000423336	COX6A1P2	17.971	30.636	59.203	25.421	0.77	7.11E-02	0.63	1.72	1.76E-06	0.9	0.50	2.63E-01	0.62
ENST00000504124	PHB	17.401	14.231	0.010	37.174	-0.29	5.80E-01	0.59	-10.77	5.80E-06	1.0	1.10	7.42E-03	0.91
ENST00000552128	TSPAN8	12.439	37.525	32.793	47.652	1.59	3.36E-04	0.93	1.40	2.37E-03	0.9	1.94	3.47E-06	0.93
ENST00000446260	C1orf122	12.039	26.796	33.197	29.131	1.15	1.82E-02	0.91	1.46	1.55E-03	0.9	1.28	7.71E-03	0.91
ENST00000598274	AC136297.1	11.912	30.379	9.581	9.989	1.35	4.42E-03	0.91	-0.31	6.24E-01	0.6	-0.25	6.89E-01	0.60
ENST00000245138	CNOT1	9.160	15.015	2.339	22.066	0.71	2.43E-01	0.62	-1.97	4.92E-02	0.9	1.27	2.15E-02	0.91
ENST00000547899	AC225613.4	9.133	19.653	34.076	23.764	1.11	5.20E-02	0.90	1.90	1.15E-04	0.9	1.38	1.08E-02	0.92
ENST00000535397	SUGT1	8.120	15.407	0.314	18.455	0.92	1.40E-01	0.63	-4.69	6.80E-03	1.0	1.18	4.70E-02	0.91
ENST00000330978	BOLA2	7.905	21.649	48.823	22.639	1.45	1.16E-02	0.91	2.63	1.54E-08	1.0	1.52	7.64E-03	0.92
ENST00000598979	BCL2L12	7.795	15.094	19.981	18.147	0.95	1.34E-01	0.63	1.36	2.14E-02	0.9	1.22	4.41E-02	0.91
ENST00000599974	AC244163.2	6.624	14.306	69.706	17.204	1.11	9.88E-02	0.90	3.40	1.22E-14	1.0	1.38	3.16E-02	0.91
ENST00000522521	MRPL15	6.408	10.840	31.533	17.194	0.76	2.99E-01	0.62	2.30	2.88E-05	1.0	1.42	2.75E-02	0.91
ENST00000444743	TIMM23	6.398	19.608	15.796	24.905	1.62	9.63E-03	0.93	1.30	4.86E-02	0.9	1.96	8.13E-04	0.93
ENST00000409905	AC106876.2	4.049	11.407	8.315	14.092	1.49	6.64E-02	0.91	1.04	2.42E-01	0.9	1.80	1.91E-02	0.93
ENST00000553330	RP11-187E13.1	3.813	7.510	11.871	7.656	0.98	2.92E-01	0.62	1.64	4.51E-02	0.9	1.01	2.76E-01	0.89
ENST00000517619	SPIDR	2.982	9.033	0.844	14.152	1.60	8.97E-02	0.92	-1.82	3.33E-01	0.9	2.25	6.83E-03	0.98
ENST00000507286	HSD17B11	2.335	1.022	17.757	2.350	-1.19	5.37E-01	0.87	2.93	4.14E-04	1.0	0.01	9.95E-01	0.59
ENST00000457288	CBWD7	2.212	7.223	11.331	7.435	1.71	1.17E-01	0.92	2.36	1.37E-02	1.0	1.75	1.05E-01	0.92
ENST00000588563	METTL23	2.112	3.657	11.997	4.884	0.79	5.57E-01	0.60	2.51	8.44E-03	1.0	1.21	3.28E-01	0.89
ENST00000599234	AC013449.1	1.877	2.710	14.909	3.652	0.53	7.29E-01	0.58	2.99	1.17E-03	1.0	0.96	4.91E-01	0.62
ENST00000400061	LYPLA2	1.669	14.974	25.204	13.361	3.17	8.33E-04	0.99	3.92	1.45E-06	1.0	3.00	2.19E-03	0.99
ENST00000487284	CUX1	1.324	5.251	2.394	7.752	1.99	1.49E-01	0.91	0.86	6.29E-01	0.6	2.55	3.71E-02	0.97
ENST00000506424	ANKRD37	1.311	1.793	7.607	2.070	0.45	8.17E-01	0.58	2.54	3.98E-02	1.0	0.66	7.23E-01	0.59
ENST00000449624	COASY	1.264	5.843	2.342	13.006	2.21	1.02E-01	0.96	0.89	6.22E-01	0.6	3.36	1.49E-03	0.99
ENST00000458112	GNPDA1	0.985	0.684	7.239	1.313	-0.53	8.60E-01	0.58	2.88	3.33E-02	1.0	0.41	8.62E-01	0.59
ENST00000269194	C18orf21	0.953	4.978	0.010	7.479	2.39	1.21E-01	0.96	-6.57	5.17E-01	0.8	2.97	2.76E-02	0.98
ENST00000425276	MTHFD1L	0.713	6.774	11.428	0.476	3.25	3.07E-02	0.99	4.00	1.62E-03	1.0	-0.59	8.79E-01	0.59
ENST00000366221	AL645728.1	0.662	0.695	11.402	2.611	0.07	9.84E-01	0.58	4.11	1.50E-03	1.0	1.98	3.50E-01	0.87
ENST00000443074	MTHFD1L	0.462	1.969	0.010	6.684	2.09	4.24E-01	0.91	-5.53	7.26E-01	0.6	3.85	2.24E-02	0.99
ENST00000541204	RNF4	0.456	1.929	1.818	6.759	2.08	4.32E-01	0.87	2.00	4.61E-01	0.9	3.89	2.11E-02	0.99
ENST00000558623	DISP2	0.264	0.731	5.130	0.036	1.47	7.55E-01	0.58	4.28	4.51E-02	1.0	-2.89	8.57E-01	0.59

ENST00000311469	COQ2	0.191	5.689	3.436	0.208	4.90	2.75E-02	0.98	4.17	1.23E-01	1.0	0.12	9.90E-01	0.59
ENST00000389312	NBR1	0.180	6.230	3.582	9.972	5.11	1.88E-02	0.99	4.31	1.10E-01	1.0	5.79	1.50E-03	0.99
ENST00000593068	TMEM259	0.035	7.851	6.394	3.980	7.83	4.67E-03	0.99	7.53	1.28E-02	1.0	6.85	6.72E-02	0.97
ENST00000372099	GTF3C5	0.010	14.657	11.096	12.201	10.52	3.88E-05	1.00	10.12	4.58E-04	1.0	10.25	2.13E-04	1.00
ENST00000395574	DGKZ	0.010	3.799	4.639	5.034	8.57	7.19E-02	0.97	8.86	4.02E-02	1.0	8.98	3.06E-02	0.98
ENST00000556580	NGDN	0.010	0.010	6.893	0.010	0.00	9.99E-01	0.58	9.43	8.42E-03	1.0	0.00	1.00E+00	0.59
ENST00000453009	IFT27	0.010	1.624	4.337	0.513	7.34	3.25E-01	0.91	8.76	4.95E-02	1.0	5.68	7.01E-01	0.81
ENST00000583744	ASPSCR1	0.010	7.253	0.010	5.046	9.50	6.56E-03	0.99	0.00	1.00E+00	0.6	8.98	3.03E-02	0.98
ENST00000494920	KDM1A	0.010	3.366	5.503	5.624	8.40	9.70E-02	0.96	9.10	2.21E-02	1.0	9.14	2.03E-02	0.98
ENST00000433710	MIPEP	0.010	5.959	8.886	5.334	9.22	1.61E-02	0.98	9.80	2.11E-03	1.0	9.06	2.48E-02	0.98
ENST00000547793	AC002472.1	0.010	2.322	0.843	14.983	7.86	2.00E-01	0.94	6.40	5.57E-01	0.8	10.55	3.09E-05	1.00
ENST00000366922	IARS2	0.010	8.129	0.010	10.728	9.67	3.63E-03	0.99	0.00	9.98E-01	0.6	10.07	6.01E-04	0.99
ENST00000392566	HN1	0.010	0.010	4.977	0.010	0.00	1.00E+00	0.58	8.96	3.17E-02	1.0	0.00	1.00E+00	0.59
ENST00000586283	CDC34	0.010	0.010	0.016	5.893	0.00	9.98E-01	0.58	0.71	9.89E-01	0.6	9.20	1.68E-02	0.98
ENST00000433404	SAFB	0.010	5.439	4.024	0.017	9.09	2.32E-02	0.98	8.65	6.18E-02	1.0	0.79	9.90E-01	0.59
ENST00000561457	C9orf69	0.010	5.278	0.010	0.010	9.04	2.59E-02	0.98	0.00	9.99E-01	0.6	0.00	9.98E-01	0.59
ENST00000417400	CITED1	0.010	0.010	5.200	0.010	0.00	9.99E-01	0.58	9.02	2.72E-02	1.0	0.00	1.00E+00	0.59
ENST00000395909	AURKA	0.010	4.378	0.150	1.835	8.77	4.84E-02	0.98	3.91	9.03E-01	0.6	7.52	2.81E-01	0.91
ENST00000378921	ZC3H13	0.010	2.895	0.980	8.988	8.18	1.34E-01	0.95	6.62	5.07E-01	0.8	9.81	1.97E-03	0.99
ENST00000538784	NOC4L	0.010	7.204	1.620	4.691	9.49	6.79E-03	0.99	7.34	3.26E-01	0.9	8.87	3.88E-02	0.98
ENST00000589735	LUZP6	0.010	11.781	8.640	40.187	10.20	2.84E-04	1.00	9.76	2.51E-03	1.0	11.97	8.00E-13	1.00
ENST00000545148	EXOC4	0.010	0.875	3.240	8.437	6.45	5.45E-01	0.81	8.34	1.06E-01	1.0	9.72	2.89E-03	0.99
ENST00000600947	BCL2L12	0.010	1.555	2.519	6.734	7.28	3.40E-01	0.91	7.98	1.75E-01	1.0	9.40	9.40E-03	0.99
ENST00000392079	NFKBIB	0.010	9.848	0.157	7.564	9.94	1.10E-03	0.99	3.97	9.00E-01	0.6	9.56	5.33E-03	0.99
ENST00000590621	C19orf25	0.010	0.752	5.457	1.803	6.23	5.94E-01	0.81	9.09	2.28E-02	1.0	7.49	2.87E-01	0.91
ENST00000395911	AURKA	0.010	3.504	0.010	5.237	8.45	8.81E-02	0.97	0.00	1.00E+00	0.6	9.03	2.65E-02	0.98
ENST00000439065	MYO1B	0.010	3.918	2.003	5.446	8.61	6.61E-02	0.97	7.65	2.49E-01	0.9	9.09	2.29E-02	0.98
ENST00000606391	SHC1	0.010	2.511	5.942	5.657	7.97	1.75E-01	0.95	9.22	1.63E-02	1.0	9.14	1.98E-02	0.98
ENST00000474739	ATG4B	0.010	0.261	0.010	6.812	4.71	8.37E-01	0.58	0.00	9.97E-01	0.6	9.41	8.98E-03	0.99
ENST00000546670	DCTN2	0.010	7.715	1.777	4.016	9.59	4.77E-03	0.99	7.47	2.92E-01	0.9	8.65	6.20E-02	0.98

Table S4. Novel alternative splicing events found in LIM1863 cells and released EVs.

chr_ID	start	end	strand	Cell	sMV	A33-Exos	EpCAM-Exos	Gene
1	26094705	26094785	+	0	0	120	0	MAN1C1
1	24018320	24019101	+	136	395	0	624	RPL11
1	24019250	24020300	+	181	503	1286	519	RPL11
1	24021232	24022287	+	0	901	0	1522	RPL11

1	24021277	24022285	+	0	226	0	0	RPL11
1	24021282	24022298	+	0	0	0	105	RPL11
1	6246771	6252989	-	42	135	416	191	RPL22
1	6246840	6252989	-	0	186	0	0	RPL22
1	6246877	6253006	-	37	108	0	258	RPL22
1	93301795	93301897	+	0	485	0	0	RPL5
1	93301950	93303045	+	1	0	0	227	RPL5
1	153964142	153964540	+	0	1005	0	0	RPS27
1	45242387	45243304	+	0	0	0	569	RPS8
1	45242412	45243285	+	0	0	0	383	RPS8
1	43166673	43167656	+	0	0	0	130	YBX1
1	43166718	43167656	+	3	117	242	227	YBX1
2	207025435	207026077	+	0	0	1900	0	EEF1B2
2	207025698	207026077	+	0	101	0	177	EEF1B2
2	207026162	207026759	+	0	0	0	101	EEF1B2
2	101620771	101622420	+	33	148	248	158	RPL31
2	101622477	101622821	+	0	0	0	307	RPL31
2	55461306	55461964	+	0	0	156	55	RPS27A
2	55462074	55462572	+	0	0	216	0	RPS27A
2	3622941	3623190	+	40	99	262	119	RPS7
2	3623479	3624148	+	260	610	2150	931	RPS7
2	3624221	3625301	+	0	0	0	393	RPS7
2	3625365	3627713	+	0	141	0	0	RPS7
3	40500228	40502907	+	1	0	0	819	RPL14
3	40502945	40503096	+	0	0	0	114	RPL14
3	40503153	40503437	+	0	484	3382	3	RPL14
3	23959518	23959946	+	0	0	0	126	RPL15
3	23961003	23962167	+	0	31	276	0	RPL15
3	101401748	101404661	-	2	0	742	377	RPL24
3	101404764	101405325	-	1	0	0	414	RPL24
3	52027830	52027885	-	0	144	0	0	RPL29
3	52027843	52027900	-	0	0	0	579	RPL29
3	52027867	52028055	-	0	0	0	1673	RPL29
3	52028143	52029101	-	0	570	0	856	RPL29
3	52029480	52029872	-	0	196	0	0	RPL29
3	12877698	12880921	-	0	0	0	855	RPL32
3	12880941	12881640	-	0	0	4444	0	RPL32
3	39450216	39452246	+	0	150	0	8	RPSA
3	39453553	39453767	+	18	50	954	102	RPSA
3	48481869	48481954	+	42	107	198	186	TMA7
4	2077247	2078234	-	19	103	62	139	POLN
4	39456600	39458025	-	0	0	130	13	RPL9

4	152022267	152024040	+	0	0	1350	0	RPS3A
4	152022315	152024035	+	0	201	0	0	RPS3A
5	55240628	55240695	-	0	0	0	165	IL6ST
5	55240653	55240746	-	0	0	0	263	IL6ST
5	40828054	40832543	-	101	121	0	128	RPL37
5	40832676	40834312	-	0	0	11968	0	RPL37
5	149826439	149827236	-	0	0	0	1084	RPS14
5	149826527	149827236	-	3	37	278	53	RPS14
5	81572062	81572216	-	0	0	0	468	RPS23
5	76989174	77004066	-	0	4	122	3	TBCA
6	31700110	31701612	-	0	0	0	107	CLIC1
6	74227590	74228502	-	0	1710	0	0	EEF1A1
6	74227658	74227787	-	0	689	0	0	EEF1A1
6	74228946	74229066	-	0	0	0	1402	EEF1A1
6	74229231	74229605	-	712	840	0	1150	EEF1A1
6	160211093	160211971	+	78	86	42	124	MRPL18
6	35436374	35436578	+	0	0	1074	0	RPL10A
6	35436805	35437209	+	0	0	0	278	RPL10A
6	35436805	35437212	+	0	325	0	510	RPL10A
6	133135922	133136156	+	0	182	0	248	RPS12
6	33239900	33240425	+	0	0	0	823	RPS18
6	33240433	33243618	+	60	170	927	0	RPS18
6	33240504	33243587	+	0	0	0	1088	RPS18
6	33243656	33243748	+	0	0	1057	0	RPS18
6	33243661	33243717	+	12	33	157	55	RPS18
6	33243661	33243768	+	96	314	1304	404	RPS18
6	33243844	33244167	+	37	130	432	43	RPS18
8	144662876	144663223	-	0	124	0	0	EEF1D
8	144663317	144663398	-	1	0	0	103	EEF1D
8	109214153	109215218	-	3	0	0	163	EIF3E
8	99054079	99057170	-	0	0	0	103	RPL30
8	99057311	99057574	-	2	3	2478	0	RPL30
8	74203868	74204020	-	0	0	0	233	RPL7
8	74204632	74204923	-	53	0	0	147	RPL7
8	146015312	146015753	-	0	939	0	1105	RPL8
8	146015812	146016661	-	0	0	0	239	RPL8
8	146016881	146017166	-	88	188	278	30	RPL8
8	146017189	146017238	-	0	0	0	309	RPL8
8	146017300	146017382	-	0	0	0	224	RPL8
8	146017300	146017407	-	0	0	0	521	RPL8
8	56985834	56986265	-	0	0	0	1235	RPS20

9	131009766	131010861	+	100	49	14	15	DNM1
9	130213063	130213563	-	0	0	0	1024	RPL12
9	130213068	130213569	-	0	0	148	0	RPL12
9	127622506	127623762	-	0	338	0	527	RPL35
9	136216908	136217097	+	0	0	2228	0	RPL7A
9	136217175	136217511	+	0	506	0	0	RPL7A
9	19376384	19376530	-	0	153	0	0	RPS6
9	19376588	19378365	-	118	45	1258	67	RPS6
9	19376650	19378390	-	12	29	4018	34	RPS6
9	19378432	19378868	-	0	176	0	0	RPS6
9	19378510	19378705	-	0	1	0	118	RPS6
9	19379598	19380187	-	57	120	408	184	RPS6
9	113013145	113013711	-	51	195	0	208	TXN
9	113013731	113018691	-	0	137	0	144	TXN
10	79795443	79796966	+	0	0	138	0	RPS24
11	62327300	62327540	-	4	181	284	233	MIR3654
11	8705588	8706272	+	0	0	0	182	RPL27A
11	810040	811596	+	48	190	370	317	RPLP2
11	812634	812762	+	119	464	2018	610	RPLP2
11	118888253	118888667	-	0	0	0	692	RPS25
12	105742405	105760394	+	36	91	116	110	C12orf75
12	6645707	6645903	+	0	129	230	154	GAPDH
12	53689726	53691828	+	5	19	108	29	PFDN5
12	131357163	131360277	+	0	0	338	0	RAN
12	56510584	56511305	+	0	0	0	1385	RPL41
12	56510648	56510972	+	127	407	782	518	RPL41
12	112843717	112846382	-	0	0	0	375	RPL6
12	112844145	112844550	-	0	0	0	352	RPL6
12	112844607	112846043	-	0	0	0	301	RPL6
12	112846134	112846223	-	0	238	0	0	RPL6
12	120635233	120636356	-	0	0	0	586	RPLP0
12	120636438	120636656	-	2	823	0	0	RPLP0
12	120636531	120636656	-	0	1	0	566	RPLP0
12	125396391	125398214	-	0	0	1216	0	UBC
12	125396727	125398094	-	3	4	666	0	UBC
12	125396955	125398094	-	2	4	646	2	UBC
12	125397183	125398094	-	4	3	494	0	UBC
12	125397411	125398094	-	1	3	578	1	UBC
12	125397867	125398094	-	13	8	1218	0	UBC
13	43636432	43639821	+	146	97	0	73	DNAJC15

13	45911258	45911346	-	0	39	102	75	TPT1
13	45911447	45912847	-	0	493	0	708	TPT1
13	45913694	45914136	-	0	0	0	638	TPT1
13	45914180	45914295	-	0	1904	0	3041	TPT1
13	45914185	45914300	-	0	2317	0	3609	TPT1
13	45914241	45914300	-	0	0	0	1093	TPT1
13	45914306	45914882	-	0	0	0	428	TPT1
13	45914901	45915198	-	0	2140	0	0	TPT1
15	40648311	40648376	+	56	128	506	8	PHGR1
15	40648311	40648409	+	61	180	0	13	PHGR1
15	40648311	40648442	+	106	196	526	178	PHGR1
15	40648311	40648475	+	0	0	0	265	PHGR1
15	40648368	40648432	+	43	0	164	97	PHGR1
15	69745335	69747810	+	0	0	106	0	RPLP1
15	69745360	69745988	+	0	0	0	495	RPLP1
15	69745364	69745994	+	0	0	0	120	RPLP1
15	83207116	83207630	-	0	0	0	209	RPS17L
15	85212971	85213136	-	0	0	108	0	SEC11A
16	89627429	89627641	+	0	1209	0	200	RPL13
16	89628023	89628072	+	0	0	0	1306	RPL13
16	2012608	2012736	-	142	540	0	779	RPS2
16	2012651	2012736	-	138	0	1000	45	RPS2
16	2012911	2013156	-	2	0	0	459	RPS2
17	37358693	37360338	+	0	165	0	2	RPL19
17	37006720	37008856	-	4	119	308	0	RPL23
17	8280997	8283130	-	1	0	4710	0	RPL26
17	8281011	8285460	-	25	113	76	112	RPL26
17	8285594	8286474	-	0	273	0	0	RPL26
17	16285402	16285629	+	0	16	0	100	UBB
17	16285553	16285780	+	3	134	2	9	UBB
18	47016923	47017172	-	0	0	0	277	RPL17
18	47017217	47017774	-	77	0	0	293	RPL17
18	47017277	47017774	-	0	247	0	0	RPL17
18	47017955	47018115	-	47	142	210	137	RPL17
18	19202763	19203831	+	19	102	296	127	SNRPD1
19	49468740	49468842	+	0	191	0	297	FTL
19	49469174	49469540	+	107	224	600	327	FTL
19	49993170	49993383	+	0	0	0	149	RPL13A
19	49993170	49993488	+	300	757	924	946	RPL13A
19	49993534	49993736	+	0	0	0	291	RPL13A
19	49994089	49994329	+	0	0	0	1808	RPL13A
19	17972282	17972914	+	0	0	0	393	RPL18A
19	55898032	55899297	+	2	90	292	114	RPL28

19	55898062	55899302	+	3	4	0	401	RPL28
19	55899412	55899610	+	0	491	0	0	RPL28
19	55899417	55899614	+	26	95	140	117	RPL28
19	50000553	50001274	+	0	0	7222	0	RPS11
19	50000557	50000776	+	0	145	0	177	RPS11
19	50000820	50002768	+	1	155	300	221	RPS11
19	50001304	50002778	+	3	219	1046	327	RPS11
19	39924186	39924304	-	1	0	1124	300	RPS16
19	39926346	39926487	-	98	223	1520	386	RPS16
19	58899613	58905863	+	44	85	214	144	RPS5
19	58904553	58904737	+	5	119	0	4	RPS5
19	58905963	58906075	+	0	111	374	104	RPS5
19	54704757	54705033	+	0	148	0	0	RPS9
19	54710331	54711278	+	0	0	0	251	RPS9
19	54710331	54711320	+	0	123	0	0	RPS9
20	32868457	32868832	-	216	355	166	350	AHCY
20	62152943	62153057	+	0	143	0	240	PPDPF
20	60962442	60962664	+	4	18	102	18	RPS21
20	60962731	60962907	+	0	256	0	0	RPS21
20	60962731	60963364	+	41	112	190	125	RPS21
22	39709301	39709638	-	0	0	0	195	RPL3
22	39710190	39710716	-	0	0	0	377	RPL3
22	39711537	39712710	-	0	276	0	0	RPL3
22	39714572	39715599	-	55	96	42	140	RPL3
HG1079_PATCH	54704143	54704419	+	0	149	0	0	RPS9
HG1079_PATCH	54709717	54710664	+	0	0	0	248	RPS9
HG1079_PATCH	54709717	54710706	+	0	121	0	0	RPS9
HG1350_HG959_PATCH	42367118	42374944	+	0	0	0	165	RPS19
HG1426_PATCH	77160548	77163603	+	2	0	0	106	COX7B
HG1497_PATCH	153568410	153569025	+	22	76	0	132	RPL10
HG1497_PATCH	153569095	153569184	+	82	216	969	255	RPL10
HG1497_PATCH	152964329	152964905	+	20	72	172	88	SSR4
HG79_PATCH	136225588	136225777	+	0	0	2156	0	RPL7A
HG79_PATCH	136225855	136226191	+	0	426	0	0	RPL7A
HSCHR19LRC_COX1_CTG1	54704575	54704851	+	0	116	0	0	RPS9
HSCHR19LRC_COX2_CTG1	54704757	54705033	+	0	120	0	0	RPS9
HSCHR19LRC_COX2_CTG1	54710331	54711278	+	0	0	0	209	RPS9
HSCHR19LRC_COX2_CTG1	54710331	54711320	+	0	107	0	0	RPS9
HSCHR19LRC_LRC_I_CTG1	54704757	54705033	+	0	117	0	0	RPS9
HSCHR19LRC_LRC_I_CTG1	54710331	54711278	+	0	0	0	211	RPS9
HSCHR19LRC_LRC_I_CTG1	54710331	54711320	+	0	105	0	0	RPS9
HSCHR19LRC_LRC_J_CTG1	54704757	54705033	+	0	115	0	0	RPS9

HSCHR19LRC_LRC_J_CTG1	54710331	54711278	+	0	0	0	212	RPS9
HSCHR19LRC_LRC_J_CTG1	54710331	54711320	+	0	109	0	0	RPS9
HSCHR19LRC_LRC_S_CTG1	54704757	54705033	+	0	112	0	0	RPS9
HSCHR19LRC_LRC_S_CTG1	54710331	54711278	+	0	0	0	209	RPS9
HSCHR19LRC_LRC_S_CTG1	54710331	54711320	+	0	105	0	0	RPS9
HSCHR19LRC_LRC_T_CTG1	54704757	54705033	+	0	119	0	0	RPS9
HSCHR19LRC_LRC_T_CTG1	54710331	54711278	+	0	0	0	211	RPS9
HSCHR19LRC_LRC_T_CTG1	54710331	54711320	+	0	103	0	0	RPS9
HSCHR19LRC_PGF1_CTG1	54704748	54705024	+	0	114	0	0	RPS9
HSCHR19LRC_PGF1_CTG1	54710321	54711268	+	0	0	0	213	RPS9
HSCHR19LRC_PGF1_CTG1	54710321	54711310	+	0	106	0	0	RPS9
HSCHR19LRC_PGF2_CTG1	54704757	54705033	+	0	115	0	0	RPS9
HSCHR19LRC_PGF2_CTG1	54710331	54711278	+	0	0	0	212	RPS9
HSCHR19LRC_PGF2_CTG1	54710331	54711320	+	0	102	0	0	RPS9
HSCHR6_MHC_COX	33161506	33162017	+	0	2	0	403	RPS18
HSCHR6_MHC_COX	33161506	33162031	+	0	0	0	699	RPS18
HSCHR6_MHC_COX	33162039	33165224	+	55	142	959	0	RPS18
HSCHR6_MHC_COX	33162110	33165193	+	0	0	0	924	RPS18
HSCHR6_MHC_COX	33165262	33165354	+	0	0	947	0	RPS18
HSCHR6_MHC_COX	33165267	33165323	+	10	31	160	50	RPS18
HSCHR6_MHC_COX	33165267	33165374	+	86	269	1207	346	RPS18
HSCHR6_MHC_COX	33165450	33165773	+	30	84	416	126	RPS18
HSCHR6_MHC_DBB	33217825	33218336	+	0	2	0	402	RPS18
HSCHR6_MHC_DBB	33217825	33218350	+	0	0	0	687	RPS18
HSCHR6_MHC_DBB	33218358	33221543	+	56	135	856	0	RPS18
HSCHR6_MHC_DBB	33218429	33221512	+	0	0	0	918	RPS18
HSCHR6_MHC_DBB	33221581	33221673	+	0	0	895	0	RPS18
HSCHR6_MHC_DBB	33221586	33221642	+	11	32	158	53	RPS18
HSCHR6_MHC_DBB	33221586	33221693	+	86	266	1250	350	RPS18
HSCHR6_MHC_DBB	33221769	33222092	+	30	82	397	124	RPS18
HSCHR6_MHC_MCF	29849710	29991765	+	0	224	0	0	HLA-A
HSCHR6_MHC_MCF	33410273	33410784	+	0	2	0	376	RPS18
HSCHR6_MHC_MCF	33410273	33410798	+	0	0	0	687	RPS18
HSCHR6_MHC_MCF	33410806	33413995	+	56	144	884	0	RPS18
HSCHR6_MHC_MCF	33410877	33413964	+	0	0	0	935	RPS18
HSCHR6_MHC_MCF	33414033	33414125	+	0	0	946	0	RPS18
HSCHR6_MHC_MCF	33414038	33414094	+	12	30	164	48	RPS18
HSCHR6_MHC_MCF	33414038	33414145	+	84	267	1230	352	RPS18
HSCHR6_MHC_MCF	33414221	33414544	+	30	84	407	125	RPS18
HSCHR6_MHC_QBL	33168739	33169250	+	0	2	0	381	RPS18
HSCHR6_MHC_QBL	33168739	33169264	+	0	0	0	686	RPS18
HSCHR6_MHC_QBL	33169272	33172460	+	53	147	874	0	RPS18
HSCHR6_MHC_QBL	33169343	33172429	+	0	0	0	919	RPS18

HSCHR6_MHC_QBL	33172498	33172590	+	0	0	927	0	RPS18
HSCHR6_MHC_QBL	33172503	33172559	+	12	31	161	48	RPS18
HSCHR6_MHC_QBL	33172503	33172610	+	85	268	1200	349	RPS18
HSCHR6_MHC_QBL	33172686	33173009	+	30	84	412	126	RPS18
HSCHR6_MHC_SSTO	33379244	33379755	+	0	0	0	162	RPS18
MT	6831	6880	+	864	853	0	0	MT-CO1
X	77155091	77158146	+	2	0	0	106	COX7B
X	153628283	153628898	+	17	62	0	109	RPL10
X	153628968	153629057	+	60	162	1013	184	RPL10
X	71493803	71494908	-	0	0	0	713	RPS4X
X	71493817	71494902	-	0	681	0	310	RPS4X
X	153061312	153061888	+	20	72	172	88	SSR4
X	47516685	47517180	-	0	0	126	0	UXT

Table S5. Fusion gene events found in LIM1863 cells and released EVs.

Gene_seq(5p)	Gene_seq(3p)	Genes5p	Genes3p	Type	Distance	Cell	sMVs	A33-Exos	EpCAM-Exos	Total_frags
6:34204649:34208658:+	5:180374511:180377611:+	HMGA1	BTNL8	Interchromosomal	None	3	5	4	7	19
X:122866801:122866903:-	17:4891425:4893559:-	THOC2	INCA1	Interchromosomal	None	-	-	-	30	30
8:90769974:90785055:+	8:90933307:90940095:+	RIPK2	OSGIN2	Read_Through	110795	20	-	-	-	20
9:113006298:113007395:-	4:14144915:14145215:+	TXN	RP11-669M16.2	Interchromosomal	None	-	3	-	9	12
7:152063847:152064099:+	7:56078759:56088923:-	RP11-208G20.3	PSPH	Intrachromsomal_Diverging	-95944550	-	-	11	-	11
1:164262:173861:-	1:243242289:243245426:-	RP11-34P13.13	RP11-261C10.3	Intrachromosomal_Complex	2.43E+08	9	6	-	-	15
5:180326273:180375945:+	6:34212607:34214007:+	BTNL8	HMGA1	Interchromosomal	None	8	-	-	6	14
20:55933495:55934877:-	MT:3229:3303:+	MTRNR2L3	MT-TL1	Interchromosomal	None	24	26	-	-	50
20:55933495:55934877:-	MT:3306:4261:+	MTRNR2L3	MT-ND1	Interchromosomal	None	12	21	-	-	33
13:113980233:114009750:-	5:179195852:179200951:+	GRTP1	MAML1	Interchromosomal	None	11	2	-	5	18
1:204183009:204183219:-	1:204159468:204162041:-	GOLT1A	KISS1	Read_Through	-1673	6	9	12	9	36
4:152048769:152147659:-	4:151185810:151412186:-	SH3D19	LRBA	Intrachromosomal	-538558	40	12	-	3	55
1:19436873:19437051:-	X:108297360:108297791:-	UBR4	CTD-2328D6.1	Interchromosomal	None	-	-	23	-	23
9:470290:540666:+	9:549189:549530:+	KANK1	RP11-31F19.1	Overlapping_Same	0	3	2	5	2	12
18:77724629:77730734:+	9:131479025:131483196:+	HSBP1L1	PKN3	Interchromosomal	None	-	-	21	2	23
X:122866801:122866903:-	17:4891424:4893559:-	THOC2	INCA1	Interchromosomal	None	25	32	20	-	77
7:45798672:45808616:-	7:55840873:55840980:+	SEPT7P2	PSPHP1	Intrachromsomal_Diverging	10023872	17	28	29	39	113
14:24767916:24768974:-	14:24739554:24740609:-	DHRS1	RABGGTA	Intrachromosomal	-27306	-	5	9	5	19

7:152063847:152064099:+	7:56078759:56079561:-	RP11-208G20.3	PSPH	Intrachromsomal_Diverging	-95944550	3	3	-	7	13
7:97601446:97601565:-	7:97595401:97596385:-	AC004967.7	OR7E38P	Read_Through	-1930	-	5	-	8	13
16:422415:425214:-	15:69745222:69747884:+	TMEM8A	RPLP1	Interchromosomal	None	3	4	12	-	19
7:56183707:56184092:-	7:56078759:56082863:-	NUPR1L	PSPH	Intrachromosomal	-63076	-	-	-	14	14
MT:4469:5510:+	MT:8526:9206:+	MT-ND2	MT-ATP6, MT-ND4	Intrachromosomal	3015	17	7	-	-	24
19:14544168:14544353:+	X:108297360:108297791:-	PKN1	CTD-2328D6.1	Interchromosomal	None	-	-	20	3	23
2:10262856:10269280:+	2:10281980:10351850:+	RRM2	C2orf48	Read_Through	9963	17	2	-	2	21
4:120375993:120376287:+	7:56078743:56088923:-	RP11-33B1.1	PSPH	Interchromosomal	None	4	4	-	13	21
16:18799384:18812840:-	12:113491866:113492239:+	RP11-1035H13.3,RPS15A	RPS15AP32	Interchromosomal	None	-	-	16	-	16
5:180326273:180374754:+	6:34212607:34214007:+	BTNL8	HMGA1	Interchromosomal	None	-	5	11	-	16
17:72199791:72205447:+	2:78019925:78020212:+	RPL38	AC105399.2	Interchromosomal	None	-	5	10	6	21
22:32435476:32435882:+	6:117205951:117239272:+	SC22CB-1E7.1	RFX6	Interchromosomal	None	-	2	13	-	15
8:37386614:37411700:-	8:37373927:37374538:-	RP11-150O12.1	RP11-150O12.6	Overlapping_Complex	0	3	8	-	7	18
7:152063847:152064099:+	7:55840873:55840980:+	RP11-208G20.3	PSPHP1	Intrachromosomal_Complex	-96222866	3	-	7	7	17
8:90769974:90785055:+	8:90933307:90933546:+	RIPK2	OSGIN2	Read_Through	111697	-	18	-	23	41

Table S6. RNA binding proteins identified within the three EV subtypes from LIM1863 cells.

Category	RNA target[a]	Protein Names	Protein Description	speC		
				A33-Exos	EpCAM-Exos	sMVs
	diverse	RNH1	ribonuclease/angiogenin inhibitor 1	12	6	11
	diverse	SKIV2L2	superkiller viralicidic activity 2-like 2 (S. cerevisiae)	2	3	
	mRNA	API5	apoptosis inhibitor 5	12	16	
	mRNA	BZW1	basic leucine zipper and W2 domains 1	4		3
	mRNA	BZW2	basic leucine zipper and W2 domains 2	3		7
	mRNA	C1QBP	complement component 1, q subcomponent binding protein		4	9
	mRNA	CNOT1	CCR4-NOT transcription complex, subunit 1	9		12
	mRNA	CRYZ	crystallin, zeta (quinone reductase)	6		
	mRNA	CSTF3	cleavage stimulation factor, 3' pre-RNA, subunit 3, 77kDa	24	2	
DDX	mRNA	DDX42	DEAD (Asp-Glu-Ala-Asp) box polypeptide 42	5		
	mRNA	DDX6	DEAD (Asp-Glu-Ala-Asp) box polypeptide 6	8		5
	mRNA	DEK	DEK oncogene		20	
	mRNA	DRG1	developmentally regulated GTP binding protein 1	8		9
	mRNA	DYNC1H1	dynein, cytoplasmic 1, heavy chain 1	98	110	233
EIFs	mRNA	EIF3A	eukaryotic translation initiation factor 3, subunit A	40	11	19
	mRNA	EIF3B	eukaryotic translation initiation factor 3, subunit B	3	3	4
	mRNA	EIF3C	eukaryotic translation initiation factor 3, subunit C	18	11	8
	mRNA	EIF3E	eukaryotic translation initiation factor 3, subunit E	12		8
	mRNA	EIF3H	eukaryotic translation initiation factor 3, subunit H	2		5
	mRNA	EIF3I	eukaryotic translation initiation factor 3, subunit I	7		4
	mRNA	EIF3J	eukaryotic translation initiation factor 3, subunit J	7	10	
	mRNA	EIF3K	eukaryotic translation initiation factor 3, subunit K	6	5	6
	mRNA	EIF3L	eukaryotic translation initiation factor 3, subunit L	33		25

	mRNA	EIF3M	eukaryotic translation initiation factor 3, subunit M	11	5	6
	mRNA	EIF4A1	eukaryotic translation initiation factor 4A, isoform 1	23	11	44
	mRNA	EIF4A2	eukaryotic translation initiation factor 4A, isoform 2	17	6	27
	mRNA	EIF4A3	eukaryotic translation initiation factor 4A, isoform 3		3	10
	mRNA	EIF5	eukaryotic translation initiation factor 5	7	5	11
	mRNA	EIF5A	eukaryotic translation initiation factor 5A	9	12	29
	mRNA	EIF5AL1	eukaryotic translation initiation factor 5A-like 1		11	24
ETFs	mRNA	ETF1	eukaryotic translation termination factor 1	22	11	6
	mRNA	GAPDH	glyceraldehyde-3-phosphate dehydrogenase	320	243	175
hnRNPs	mRNA	HNRNPAB	heterogeneous nuclear ribonucleoprotein A/B	11		13
	mRNA	HNRNPC	heterogeneous nuclear ribonucleoprotein C (C1/C2)		31	26
	mRNA	HNRNPCL1	heterogeneous nuclear ribonucleoprotein C-like 1		22	14
	mRNA	HNRNPD	heterogeneous nuclear ribonucleoprotein D	16		23
	mRNA	HNRNPK	heterogeneous nuclear ribonucleoprotein K	28	25	46
	mRNA	HNRNPR	heterogeneous nuclear ribonucleoprotein R	9		17
	mRNA	ILF2	interleukin enhancer binding factor 2, 45kDa	90	37	17
	mRNA	ILF3	interleukin enhancer binding factor 3, 90kDa	38	32	
IPOs	mRNA	IPO4	importin 4		5	
	mRNA	IPO5	importin 5	621	561	99
	mRNA	IPO7	importin 7	66	32	26
	mRNA	IPO9	importin 9	22	11	3
	mRNA	KPNB1	karyopherin (importin) beta 1	187	126	84
	mRNA	LUC7L	LUC7-like (S. cerevisiae)		5	
	mRNA	LUC7L2	LUC7-like 2 (S. cerevisiae)		19	
	mRNA	MRTO4	mRNA turnover 4 homolog (S. cerevisiae)		14	
	mRNA	NCBP1	nuclear cap binding protein subunit 1, 80kDa		3	
	mRNA	NUDT21	nudix (nucleoside diphosphate linked moiety X)-type motif 21	6		
	mRNA	NUTF2	nuclear transport factor 2	3		5
PABPCs	mRNA	PABPC1	poly(A) binding protein, cytoplasmic 1	34		10
	mRNA	PABPC1L	poly(A) binding protein, cytoplasmic 1-like	10		5
	mRNA	PABPC3	poly(A) binding protein, cytoplasmic 3	17		8
	mRNA	PABPC4	poly(A) binding protein, cytoplasmic 4 (inducible form)	13		
	mRNA	PABPC5	poly(A) binding protein, cytoplasmic 5	3		
	mRNA	PARK7	Parkinson disease (autosomal recessive, early onset) 7	10	2	20
	mRNA	PARP1	poly (ADP-ribose) polymerase 1	4		
PCBPs	mRNA	PCBP1	poly(rC) binding protein 1	34	26	31
	mRNA	PCBP2	poly(rC) binding protein 2	18	15	34
	mRNA	PCBP3	poly(rC) binding protein 3	12	8	
	mRNA	POLR2G	polymerase (RNA) II (DNA directed) polypeptide G	11	10	
	mRNA	PRDX1	peroxiredoxin 1	111	70	72
	mRNA	PRKDC	protein kinase, DNA-activated, catalytic polypeptide	37	23	196
	mRNA	PRPF19	PRP19/PSO4 pre-mRNA processing factor 19 homolog (S. cerevisiae)	3		
	mRNA	PTBP1	polypyrimidine tract binding protein 1	16	4	7
	mRNA	RAN	RAN, member RAS oncogene family	71	57	29
	mRNA	RANBP6	RAN binding protein 6	12	26	
	mRNA	RBM25	RNA binding motif protein 25	2	5	
RUVBL	mRNA	RUVBL1	RuvB-like 1 (E. coli)	10		29
	mRNA	RUVBL2	RuvB-like 2 (E. coli)	4		24
SFs	mRNA	SF3A3	splicing factor 3a, subunit 3, 60kDa			3
	mRNA	SF3B1	splicing factor 3b, subunit 1, 155kDa	10	6	14
	mRNA	SF3B3	splicing factor 3b, subunit 3, 130kDa	7	3	7
	mRNA	SFRS1	splicing factor, arginine/serine-rich 1		5	21
	mRNA	SFRS14	splicing factor, arginine/serine-rich 14	4		
	mRNA	SFRS3	splicing factor, arginine/serine-rich 3		3	8
	mRNA	SFRS7	splicing factor, arginine/serine-rich 7, 35kDa			5
	mRNA	PUF60	poly-U binding splicing factor 60KDa	15	7	

	mRNA	SMG1	SMG1 homolog, phosphatidylinositol 3-kinase-related kinase (C. elegans)		7	
	mRNA	SRRM1	serine/arginine repetitive matrix 1		4	
	mRNA	STRAP	serine/threonine kinase receptor associated protein	3		5
	mRNA	STRBP	spermatid perinuclear RNA binding protein	6	3	
	mRNA	SYNCRIP	synaptotagmin binding, cytoplasmic RNA interacting protein	7		31
	mRNA	TAF15	TAF15 RNA polymerase II, TATA box binding protein (TBP)-associated factor, 68kDa			7
	mRNA	TARBP1	TAR (HIV-1) RNA binding protein 1	15		
TNPOs	mRNA	TNPO1	transportin 1	3	8	5
	mRNA	TNPO2	transportin 2	3	7	5
	mRNA	TNPO3	transportin 3	38	3	
	mRNA	UBA1	ubiquitin-like modifier activating enzyme 1	94	30	85
	mRNA	UPF1	UPF1 regulator of nonsense transcripts homolog (yeast)			10
XPOs	mRNA	XPO1	exportin 1 (CRM1 homolog, yeast)	46	26	16
	mRNA	XPO7	exportin 7	7		3
	mRNA	XRCC6	X-ray repair complementing defective repair in Chinese hamster cells 6	28	14	10
	mRNA	YTHDF3	YTH domain family, member 3		2	
	ncRNA	DHX9	DEAH (Asp-Glu-Ala-His) box polypeptide 9	36	26	31
	ncRNA	MVP	major vault protein	260	49	131
	ncRNA	PARP4	poly (ADP-ribose) polymerase family, member 4	37	4	20
	ncRNA	RPP30	ribonuclease P/MRP 30kDa subunit		4	
	ncRNA	SRP14	signal recognition particle 14kDa (homologous Alu RNA binding protein)		18	
	ncRNA	SRP9	signal recognition particle 9kDa		6	
	ncRNA	XPO5	exportin 5	3		15
	ribosome	RPL10	ribosomal protein L10		11	23
	ribosome	RPL10A	ribosomal protein L10a	28	37	28
	ribosome	RPL10L	ribosomal protein L10-like		5	14
	ribosome	RPL11	ribosomal protein L11	4	9	13
	ribosome	RPL12	ribosomal protein L12	48	67	39
	ribosome	RPL13	ribosomal protein L13	11	26	23
	ribosome	RPL13A	ribosomal protein L13a		11	18
	ribosome	RPL14	ribosomal protein L14	6	18	35
	ribosome	RPL17	ribosomal protein L17	5	21	21
	ribosome	RPL18	ribosomal protein L18	6	23	22
	ribosome	RPL18A	ribosomal protein L18a		18	18
	ribosome	RPL19	ribosomal protein L19	14	21	20
	ribosome	RPL22	ribosomal protein L22	17	32	12
	ribosome	RPL23	ribosomal protein L23	4		11
	ribosome	RPL23A	ribosomal protein L23a	3	8	10
	ribosome	RPL24	ribosomal protein L24	5	16	22
	ribosome	RPL26	ribosomal protein L26		18	8
	ribosome	RPL27	ribosomal protein L27	8	9	10
	ribosome	RPL27A	ribosomal protein L27a		4	10
	ribosome	RPL28	ribosomal protein L28	8	2	10
	ribosome	RPL29	ribosomal protein L29		4	
	ribosome	RPL30	ribosomal protein L30	19	27	16
	ribosome	RPL31	ribosomal protein L31	4	9	6
	ribosome	RPL32	ribosomal protein L32	5	7	11
	ribosome	RPL34	ribosomal protein L34	3	6	6
	ribosome	RPL35	ribosomal protein L35	9	21	9
	ribosome	RPL35A	ribosomal protein L35a		6	9
	ribosome	RPL36	ribosomal protein L36		17	3
	ribosome	RPL36A	ribosomal protein L36a		14	5
	ribosome	RPL36AL	ribosomal protein L36a-like		16	6
	ribosome	RPL37	ribosomal protein L37	3	11	
	ribosome	RPL37A	ribosomal protein L37a		6	11
	ribosome	RPL38	ribosomal protein L38		6	12

	ribosome	RPL4	ribosomal protein L4	4		53
	ribosome	RPL5	ribosomal protein L5	4	59	14
	ribosome	RPL6	ribosomal protein L6	28	46	58
	ribosome	RPL7	ribosomal protein L7	10	41	35
	ribosome	RPL7A	ribosomal protein L7a	11	25	36
	ribosome	RPL8	ribosomal protein L8	6	14	20
	ribosome	RPL9	ribosomal protein L9	5		17
	ribosome	RPLP0	ribosomal protein, large, P0	126	94	49
	ribosome	RPS10	ribosomal protein S10	18		
	ribosome	RPS11	ribosomal protein S11	13	7	13
	ribosome	RPS12	ribosomal protein S12	23	23	12
	ribosome	RPS13	ribosomal protein S13	39	15	18
	ribosome	RPS14	ribosomal protein S14	6		16
	ribosome	RPS15A	ribosomal protein S15a	26	22	22
	ribosome	RPS16	ribosomal protein S16	48	33	32
	ribosome	RPS17	ribosomal protein S17	3	47	16
	ribosome	RPS18	ribosomal protein S18	45	21	33
	ribosome	RPS19	ribosomal protein S19	21		9
	ribosome	RPS2	ribosomal protein S2	27	31	21
	ribosome	RPS20	ribosomal protein S20	21	14	8
	ribosome	RPS23	ribosomal protein S23	8	10	13
	ribosome	RPS24	ribosomal protein S24	8	15	12
	ribosome	RPS25	ribosomal protein S25	14	22	10
	ribosome	RPS26	ribosomal protein S26		5	
	ribosome	RPS27A	ribosomal protein S27a	125	172	97
	ribosome	RPS29	ribosomal protein S29	5		
	ribosome	RPS3	ribosomal protein S3	76	52	44
	ribosome	RPS3A	ribosomal protein S3A	15	8	18
	ribosome	RPS4X	ribosomal protein S4, X-linked	41	32	41
	ribosome	RPS4Y1	ribosomal protein S4, Y-linked 1	5	6	10
	ribosome	RPS4Y2	ribosomal protein S4, Y-linked 2	6	8	8
	ribosome	RPS5	ribosomal protein S5	45	36	55
	ribosome	RPS6	ribosomal protein S6	11	18	23
	ribosome	RPS7	ribosomal protein S7	26	37	21
	ribosome	RPS8	ribosomal protein S8	6	27	31
	ribosome	RPS9	ribosomal protein S9	16	28	32
	ribosome	RPSA	ribosomal protein SA	25	16	36
	ribosome	UBA52	ubiquitin A-52 residue ribosomal protein fusion product 1	125		96
	rRNA	NPM1	nucleophosmin (nucleolar phosphoprotein B23, numatrin)		6	16
	rRNA	PA2G4	proliferation-associated 2G4, 38kDa	12		20
	rRNA	REXO4	REX4, RNA exonuclease 4 homolog (S. cerevisiae)		15	
	rRNA	SBDS	Shwachman-Bodian-Diamond syndrome	2		3
	snoRNA	DKC1	dyskeratosis congenita 1, dyskerin	4	4	8
	snoRNA	NCL	nucleolin	5		20
	snRNA	LSM6	LSM6 homolog, U6 small nuclear RNA associated (S. cerevisiae)	3		2
	snRNA	PRPF40A	PRP40 pre-mRNA processing factor 40 homolog A (S. cerevisiae)		8	
	snRNA	PRPF8	PRP8 pre-mRNA processing factor 8 homolog (S. cerevisiae)		6	19
	snRNA	SNRNP200	small nuclear ribonucleoprotein 200kDa (U5)	3		24
	snRNA	SNRNP70	small nuclear ribonucleoprotein 70kDa (U1)		45	
	snRNA	SNRPA	small nuclear ribonucleoprotein polypeptide A	14	11	6
	snRNA	SNRPB	small nuclear ribonucleoprotein polypeptides B and B1	7	8	13
	snRNA	SNRPB2	small nuclear ribonucleoprotein polypeptide B"	8	10	
	snRNA	SNRPD1	small nuclear ribonucleoprotein D1 polypeptide 16kDa	4		
	snRNA	SNRPD2	small nuclear ribonucleoprotein D2 polypeptide 16.5kDa	5		
	snRNA	SNRPD3	small nuclear ribonucleoprotein D3 polypeptide 18kDa	8	7	4
	snRNA	U2AF2	U2 small nuclear RNA auxiliary factor 2			6
	snRNA	SNRPE	small nuclear ribonucleoprotein polypeptide E	4	6	8

	tRNA	CARS	cysteinyl-tRNA synthetase	9	4	2
	tRNA	DARS	aspartyl-tRNA synthetase	10		25
	tRNA	EEF1A1	eukaryotic translation elongation factor 1 alpha 1	107	32	209
	tRNA	EEF1A2	eukaryotic translation elongation factor 1 alpha 2	60	14	109
	tRNA	EEF1D	eukaryotic translation elongation factor 1 delta	13		27
	tRNA	EEF1G	eukaryotic translation elongation factor 1 gamma	80	37	72
	tRNA	EEF2	eukaryotic translation elongation factor 2	291	133	204
	tRNA	EFTUD2	elongation factor Tu GTP binding domain containing 2	7	4	7
	tRNA	EIF2S1	eukaryotic translation initiation factor 2, subunit 1 alpha, 35kDa	6		
	tRNA	EIF2S3	eukaryotic translation initiation factor 2, subunit 3 gamma, 52kDa	19	3	6
	tRNA	EIF5B	eukaryotic translation initiation factor 5B		4	
	tRNA	EPRS	glutamyl-prolyl-tRNA synthetase	15		32
	tRNA	FARSA	phenylalanyl-tRNA synthetase, alpha subunit	52	6	14
	tRNA	FARSB	phenylalanyl-tRNA synthetase, beta subunit	59	42	28
	tRNA	GARS	glycyl-tRNA synthetase	15	8	29
	tRNA	IARS	isoleucyl-tRNA synthetase	43	6	66
	tRNA	KARS	lysyl-tRNA synthetase	31		15
	tRNA	NARS	asparaginyl-tRNA synthetase	48	22	36
	tRNA	QARS	glutaminyl-tRNA synthetase	16	15	9
	tRNA	RARS	arginyl-tRNA synthetase	31		25
	tRNA	SARS	seryl-tRNA synthetase	16	11	9
	tRNA	SSB	Sjogren syndrome antigen B (autoantigen La)	9		15
	tRNA	TARS	threonyl-tRNA synthetase	13	3	26
	tRNA	TRMT6	tRNA methyltransferase 6 homolog (S. cerevisiae)	3	23	
	tRNA	WARS	tryptophanyl-tRNA synthetase	108	61	7
	tRNA	XPOT	exportin, tRNA (nuclear export receptor for tRNAs)	16	24	14
	unknown	HRSP12	heat-responsive protein 12	9	3	8
	unknown	PSMA1	proteasome (prosome, macropain) subunit, alpha type, 1	9		13
	unknown	PSMA6	proteasome (prosome, macropain) subunit, alpha type, 6	12	12	76
	unknown	TOP1	topoisomerase (DNA) I		4	

Table S7. qRT-PCR primers.

#No.	Gene	Forward primer	Reverse primer	Tm (°C)
1	BCL7C	CAAGGATGACATCAAGAAG	AAGATACGAAGGGAAGTG	55.9
2	CKS1B	ATCTGATGTCTGAATCTGAAT	GATGTGAGGTTCTGGTTC	control
3	EEF1G	CTTACTTGAAGACGAGGA	CTTATAGAGCCACAACAG	55.9
4	GAPDH	AAAGGGTCATCATCTCTG	GCTGTTGTCATACTTCTC	control
5	MLF2	GATGAATGACATGATTGGA	TATTGGAGTAGGAGATGAC	control
6	RAB13	TTCAACAACACTTACATC	AGTAGGCAGTAGTTATTG	59.5
7	RPS3	TATCCAAGAAGAGGAAGTT	ACATTCTGTGTTCTCAAC	55.9
8	SCARB1	GCCAAGAGAAATGCTATT	TCATCAGGGATTCAGAATA	53
9	SCD	AGGAGATAAGTTGGAGAC	GTAGCAGAGACATAAGGA	53
10	TPT1	GAAGAACAGAGACCAGAA	GTCCAATAGAGCAACCAT	53
